# Directing Cation Coordination and Phase in Nickel Sulfide Nanocrystals through the Addition of Phosphines

**DOI:** 10.1021/acs.chemmater.5c02148

**Published:** 2025-12-29

**Authors:** Emma J. Endres, Yiming Chen, De-en Jiang, Janet E. Macdonald

**Affiliations:** a Department of Chemistry, 5718 Vanderbilt University, Nashville, Tennessee 37235, United States; b Vanderbilt Institute for Nanoscale Science and Engineering, Nashville, Tennessee 37235, United States; c Department of Chemical and Biomolecular Engineering, 5718 Vanderbilt University, Nashville, Tennessee 37235, United States

## Abstract

In nanocrystal syntheses, multiple routes to achieve phase control have been explored, such as precursor reactivity and cation exchange. While these routes have opened doors to phase manipulation, the former does not directly target the product structure and the latter has limitations on which structures can be formed. Here, we combine both ideas, focusing on the structure and also influencing precursor reactivity. For the first time, we intentionally used concepts from coordination chemistry to influence the metal in solution and, in turn, affect the interstitial sites that the cation fills in the solid. Through the addition of bidentate and monodentate phosphine ligands of varying bite angles, steric bulk, and electron donation ability, we were able to influence the coordination around the nickel ion and thus the product nickel sulfide phase. We discovered that the steric bulk of the phosphine had the biggest influence on the resulting product by destabilizing surfaces with highly coordinated nickel atoms by occupying coordination sites on the metal. By varying which phosphine ligand was added, we were able to selectively target pure millerite (NiS), heazlewoodite (Ni_3_S_2_), and godlevskite (Ni_9_S_8_).

## Graphical abstract

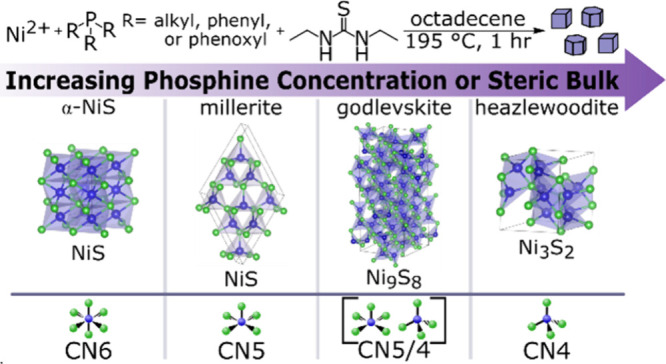

## Introduction

Being able to manipulate a colloidal reaction to produce specific phase-pure metal chalcogenides will reveal new applications as well as better current ones. In colloidal syntheses, molecular precursors decompose and then come together to form solid nanocrystals, and in this process, we have little control over the 3D crystalline structures that result. There is currently minimal ability to manipulate phases as these mechanisms are not fully understood, and many products are discovered serendipitously. There have been efforts to manipulate the phase in the synthetic routes to nickel sulfide, but most have focused on changing precursor reactivity as opposed to directly targeting structural features in the product phases.

Most commonly, researchers have altered the chalcogenide chemistry to influence the product phase. Hendricks *et al.* created a library of thiourea precursors with different reactivities, and other groups have used these to affect the phase produced for multiple metal sulfides.^1–4^ Barim *et al.* utilized two of these disubstituted thioureas, with and without the inclusion of 1-dodecanethiol as a reactivity-directing agent, while also changing the reaction temperature to access four different phases of the nickel sulfides.^2^ Espano *et al.* and Edwards *et al.* utilized this library of thioureas and different reaction temperatures to explore the iron sulfides and cobalt sulfides, respectively.^3,4^ Both works found that the iron and cobalt sulfide phases are related by their anion stackings (hcp vs ccp), allowing access to more phase-pure products by adjusting another reaction variable, the M:S ratio. Other precursor variations include using an assortment of organosulfur precursors with different bond dissociation energies^5^ and varying organophosphate reactivity to affect the composition and phase evolution of nickel phosphides.^6^

Manipulating precursor reactivity to indirectly influence the product phase has not been limited to the anions, as other studies have focused on varying the chemistry of the metal precursors. Gendler *et al.* used different manganese halides to form two phases of manganese sulfides based on a hard–soft acid–base theory.^7^ Similarly, Penk *et al.* used a variety of metal precursors and formed different metal tellurides depending on the electron chemistry of the metal precursor.^8^ Again, changing the cation precursor reactivity does not directly target specific structural features in the product phase.

Cation exchange reactions, however, do intentionally target structural features, such as anion stacking patterns, of the product phases, because the structural features of the host materials act as templates for the product. For example, several groups have obtained rare polymorphs through cation exchange by starting with template phases that contain a desired structural feature. In this way, unnatural hexagonal CuFeS_2_ that retained the structure feature of the hcp anion sublattice of the Cu_2_S host was prepared.^9^ Wurtzite CoS and MnS have been prepared from Cu_2_S because they retained both the hcp anion stacking and the tetrahedral interstitial site filling of the host.^10^ However, cation exchange has limitations; many cation exchange methods use a copper chalcogenide template because of the inherent high mobility of copper ions.^11–17^ The desired structure may not be reached from a copper chalcogenide template if the incoming cation is too large. Hernández-Pagán *et al.* found that when a pseudohexagonal copper sulfide lattice was exchanged with gold, an anion rearrangement to body-centered cubic occurred.^16^ Gariano *et al.* performed a cation exchange on cubic and hexagonal copper selenide with lead and found both converted to a rock salt crystal structure.^15^ Even with noncopper templates, this problem is still observed. Son *et al.* used cadmium selenide and performed an exchange with silver, resulting in particles with a shift in structure. Changes in the morphology were also noted when CdSe rods were used as a template.^18^ While cation exchange has allowed for some control over specific structures and morphologies, there are still limitations.

In an ideal colloidal synthesis, we could achieve phase purity using a single-step synthesis while also templating specific structural features of the phase, similar to that of cation exchange. Solution molecular chemistry has long known how to influence the structure and coordination around metal centers by using ligands. Phosphines are particularly useful tools in this regard; they are commercially available in a large variety, with well characterized and tabulated Tolman parameters to quantify their electronic donation and cone angles to measure their steric bulk.^20^ Phosphines can influence coordination number and geometry around the metal center, which are structural features that we seek to manipulate in solid nanocrystalline phases.

In this work, we target the synthesis of the nickel sulfides as they show great structural complexity. With that complexity comes disparate properties and potential applications, especially as nanocrystals. Several of the nickel sulfides show plasmonic behavior in the visible regime and are potential replacements for more expensive Ag and Au in sensing, fluorescence enhancement, etc.^21–23^ Heazlewoodite, vaesite, and α-NiS are electrocatalysts for hydrogen evolution reactions (HER).^24,25^ Heazlewoodite, α-NiS, and millerite are electroactive materials for supercapacitors.^26–29^ Millerite is a semiconductor, while heazlewoodite is a metallic conductor.^30,31^ Most of the nickel sulfides are antiferromagnets and show size dependent onset of paramagnetism.^32,33^ Application of these behaviors to commercial products can be achieved only if each of the phases can be selectively synthesized.

It is difficult to rationally predict a route to each of the nickel sulfides. Most transition metal chalcogenides can be described by anion close packing, as mostly hexagonal close packing (hcp) or cubic close packing (ccp), with the cations filling tetrahedral and/or octahedral interstitial sites. The nickel sulfides defy these simplistic descriptions, with seven different phases with different cation site filling and stoichiometries (Figure 1). Vaesite (NiS_2_) and α-NiS (NiS) have octahedral interstitial site filling (O_h_, CN6), and heazlewoodite (Ni_3_S_2_) has distorted tetrahedral cation coordination (T_d_, CN4). The oddity of this system is the existence of square pyramidal sites (Sq.Py, CN5) seen in millerite (NiS). The other nickel sulfides have mixed cation sites: godlevskite (Ni_9_S_8_) and α-Ni_7_S_6_ (Ni_7_S_6_) contain T_d_ and Sq.Py. sites, while polydymite (Ni_3_S_4_) has a combination of O_h_ and T_d_ sites. The multiple types of cation coordination indicate that the phases might be particularly sensitive to precursor coordination in the solution phase before and as they become part of the growing nanocrystals. The nickel sulfides also have varying cation oxidation states, anion packing, and polyhedral configurations (Figure 1). Throughout the paper, we focus on the changes in cation site coordination, which in turn change the stoichiometry and cause shifts in the other phase descriptors.

**Figure 1:**
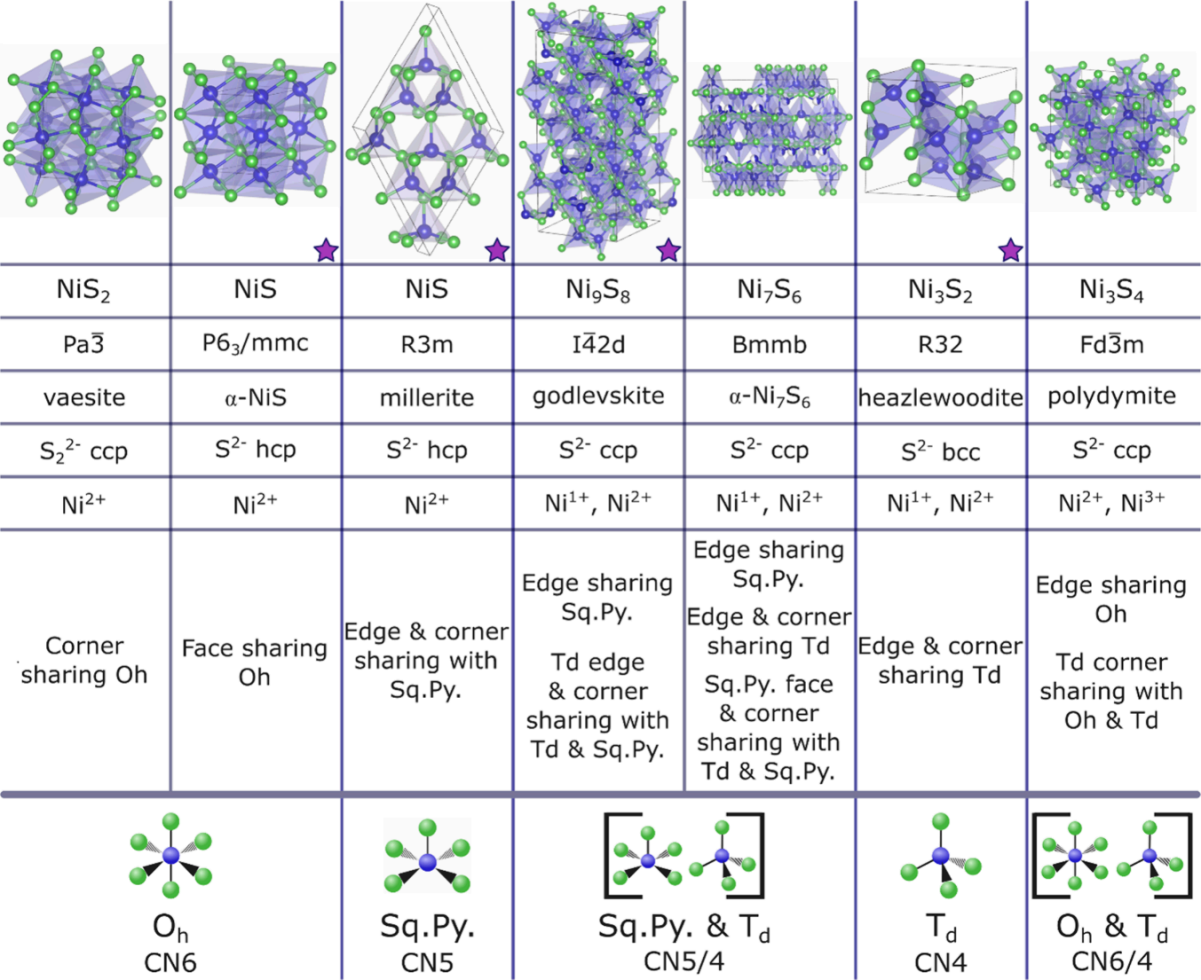
Nickel sulfide phases ordered by interstitial site filling by cations (from left to right), octahedral (O_h_, CN6): vaesite and α-NiS; square pyramidal (Sq.Py., CN5): millerite; Sq.Py. and tetrahedral (T_d_, CN4): godlevskite and α-Ni_7_S_6_; T_d_: heazlewoodite; and O_h_ and T_d_: polydymite. The phase stoichiometry, space group, phase name, anion structure, cation oxidation state, and polyhedra sharing are included for each crystal structure. The phases synthesized in this study are marked with purple star. Crystal structures were made in VESTA.^19^

Varying routes to these different phases of nickel sulfides have been reported. Many of the studies do not achieve phase control broadly across the phase space or produce phase-pure products.^25,30,33,34^ A common route in colloidal and solvothermal syntheses is to vary the ratio of the nickel and sulfur precursors to produce different phases—this method has produced pure α-NiS,^24,32^ polydymite (Ni_3_S_4_),^32^ heazlewoodite (Ni_3_S_2_),^24^ and vaesite (NiS_2_).^24^ The high-temperature phase, α-Ni_7_S_6_, can only be accessed above ∼400 °C and has been directly formed synthetically as well as from phase transitions from godlevskite (Ni_9_S_8_) and millerite (NiS).^35^ Specific studies to understand the phase control of the nickel sulfides include Barim *et al.*, which accessed phase-pure α-NiS, polydymite (Ni_3_S_4_), heazlewoodite (Ni_3_S_2_), and godlevskite (Ni_9_S_8_) by utilizing two different thiourea sulfur precursors, the addition of 1-dodecanethiol as a reactivity-directing agent, a range of reaction temperatures, and varied nickel to sulfur ratios.^2^ Roffey *et al.* performed a similar study with a single source precursor, with and without the inclusion of an additional sulfur source, tetra-iso-butyl thiuram disulfide, at varying ratios and reaction temperatures; they synthesized pure millerite (NiS) and α-NiS, as well as phase mixtures that included vaesite (NiS_2_) and polydymite (Ni_3_S_4_).^36^ Throughout these studies, the main parameter that has been demonstrated to control the phase outcome is the nickel-to-sulfur ratio, with little exploration of the positions either ion is adopting within the produced crystal.

Here and for the first time, we use coordination chemistry concepts in solution to affect the coordination that the metal assumes in the solid. Through utilization of a collection of monodentate and bidentate phosphine ligands, we evaluate how different bite angles, steric bulk, and electronic properties affect the metal coordination in the resulting nanoparticle phase. In this manner, heazlewoodite, godlevskite, and millerite were selectively prepared in a colloidal synthesis. The steric bulk of the phosphine was shown to have the most influence on the product coordination by taking up coordination sites on the metal. Evidence supports that the phosphines influence the phase both at the nucleation stage and through surface effects on larger particles.

## Materials and Methods

All syntheses were performed in oven-dried three-neck round-bottom flasks using standard Schlenk techniques under a nitrogen atmosphere. A thermocouple was used to monitor the internal temperature of the reaction (Figure S1).

### Materials

The following chemicals were purchased from Sigma-Aldrich: 1,3-bis­(diphenylphosphino)­propane (97%), 1-octadecene (technical grade, 90%), benzene-*d*_6_ (99.6%), celite, chloroform-*d* (99.8%), sulfur (powder), tri­(*o*-tolyl)­phosphine (97%), tributylphosphine (mixture of isomers, 97%), and trioctylphosphine (97%). Nickel­(II) stearate was purchased from AmBeed, Inc. Tri­(*p*-tolyl)­phosphine (98%) was purchased from Ark Pharm, Inc. Tricyclohexylphosphine (97%) and triphenylphosphite (97%) were purchased from Strem Chemicals. Ethyldiphenylphosphine (>97%) and *N*,*N*-diethylthiourea (>97%) were purchased from TCI Chemicals. Dimethylphenylphosphine (97%) was purchased from AA Blocks, and 9,9-dimethyl-4,5-bis­(di-*tert*-butylphosphino)­xanthene (97%) was purchased from Oakwood Chemical. All materials were used from the commercial suppliers without further purification.

### General Synthesis of Nickel Sulfide Nanoparticles

Nickel­(II) stearate (Ni­(C_18_H_37_CO_2_)_2_, 0.5 mmol), the desired phosphine (0.25 mmol-4 mmol), and 1-octadecene (ODE, 10 mL) were added to a 25 mL three-neck round-bottom flask. *N*,*N*-Diethylthiourea (0.5 mmol-8 mmol) and ODE (5 mL) were added to an attached, pressure-equilibrized addition funnel fitted with a thermocouple. The apparatus was placed under vacuum, while the round bottom was heated to 100 °C and held for 30 min. After refilling with nitrogen, the round bottom was heated to 195 °C, and then the addition funnel was heated to 195 °C with a heat gun. Once at the temperature, the contents of the addition funnel were swiftly released into the reaction flask. The reaction was held at a constant temperature and continuously stirred for 1 h. Finally, the reaction was pulled off heat and cooled to room temperature. The nanoparticles were isolated by precipitation with ethanol and centrifuged at 8700 rpm for 5 min. Then, the nanoparticles were resuspended in chloroform. This process was repeated two more times.

Throughout these syntheses, varying phosphine ligands were employed, in Ni:P ratios of 1:1, 1:2, and 1:4. The collection includes ethyldiphenylphosphine (PEtPh_2_), triphenylphosphite (P­(OPh)_3_), tricyclohexylphosphine (PCy_3_), tributylphosphine (PBu_3_), trioctylphosphine (TOP), tri­(*p*-tolyl)­phosphine (P­(*p*-tol)_3_), tri­(*o*-tolyl)­phosphine (P­(*o*-tol)_3_), dimethylphenylphosphine (PMe_2_Ph), 1,3-bis­(diphenylphosphino)­propane (dppp), and 9,9-dimethyl-4,5-bis­(di-*tert*-butylphosphino)­xanthene (xantphos). Dppp and xantphos are bidentate, with bite angles of 91 and 108°, respectively.^37^ The concentration of the *N*,*N*-diethylthiourea was also adjusted to study the relationship between the phosphine and thiourea concentrations, and these ratios include Ni:S—1:1, 1:2, 1:4, 1:6, 1:8, and 1:16.

### Dppp Sulfide Synthesis

Synthesis of 1,3-bis­(diphenylphosphino)­propane sulfide was adapted from Breshears *et al.* ^38^ A 6-dram vial was loaded with elemental sulfur (0.25 mmol) and toluene (10 mL), and a second vial was loaded with dppp (1 mmol). The toluene/sulfur was added to the phosphine vial and stirred for 48 h. The solution was then filtered over celite, and the solvent was removed under vacuum leaving a white solid. ^31^P­{^1^H} NMR δ (ppm): 43.1.

### NMR-Scale dppp and *N*,*N*-Diethylthiourea Control

Dppp (0.005 mmol) and *N*,*N*-diethylthiourea (0.03 mmol) were loaded into an NMR tube. Subsequently, the NMR tube was loaded into the glovebox, ODE (150 μL) was added, and then it was sealed with a septum. Once removed from the glovebox, the NMR tube was placed into a preheated oil bath (195 °C) with an N_2_-filled balloon affixed to the top. The reaction mixture was heated for 60 min. Upon cooling, 600 μL of CDCl_3_ was added, and ^31^P NMR was taken. ^31^P­{^1^H} NMR δ (ppm): 16.29. A control of only dppp in the ODE was performed in the same manner. ^31^P­{^1^H} NMR δ (ppm): 16.29.

### Surface Effect Study with Nanoparticles as Reagents

#### α-NiS Particles

Nickel­(II) stearate (Ni­(C_18_H_37_CO_2_)_2_, 0.5 mmol) and 1-octadecene (ODE, 10 mL) were added to a 25 mL three-neck round-bottom flask. *N*,*N*-Diethylthiourea (3 mmol) and ODE (5 mL) were added to an attached pressure-equilibrated addition funnel fitted with a thermocouple. The apparatus was placed under vacuum, while the round bottom was heated to 100 °C and held for 30 min. After refilling with nitrogen, the round bottom was heated to 195 °C, and then the addition funnel was heated to 195 °C with a heat gun. Once at temperature, the contents of the addition funnel were swiftly released into the reaction flask. The reaction was held at a constant temperature and continuously stirred for 1 h. Finally, the reaction was pulled off heat and allowed to cool to room temperature. The nanoparticles were isolated by precipitation with ethanol and centrifuged at 8700 rpm for 5 min. Then, the nanoparticles were resuspended in approximately 5 mL of chloroform. This process was repeated two more times.

#### Millerite Particles

Nickel­(II) stearate (Ni­(C_18_H_37_CO_2_)_2_, 0.5 mmol), dppp (0.25 mmol), and 1-octadecene (ODE, 10 mL) were added to a 25 mL three-neck round-bottom flask. *N*,*N*-Diethylthiourea (3 mmol) and ODE (5 mL) were added to an attached pressure-equilibrized addition funnel fitted with a thermocouple. The apparatus was placed under vacuum, while the round bottom was heated to 100 °C and held for 30 min. After refilling with nitrogen, the round bottom was heated to 195 °C, and then the addition funnel was heated to 195 °C with a heat gun. Once at temperature, the contents of the addition funnel were swiftly released into the reaction flask. The reaction was held at a constant temperature and continuously stirred for 1 h. Finally, the reaction was pulled off heat and allowed to cool to room temperature. The nanoparticles were isolated by precipitation with ethanol and centrifuged at 8700 rpm for 5 min. Then, the nanoparticles were resuspended in approximately 5 mL of chloroform. This process was repeated two more times.

#### Surface Effect Study

All particles from the above synthesis, the desired phosphine (PEtPh_2_ or P­(OPh)_3_, 1 mmol), and 1-octadecene (ODE, 10 mL) were added to a 25 mL three-neck round-bottom flask. The apparatus was placed under vacuum and left at room temperature until boiling of chloroform stopped. Then, the round bottom was heated to 100 °C and held for 30 min. After refilling with nitrogen, the round bottom was heated to 195 °C. The reaction was held at a constant temperature and continuously stirred for 1 h. Finally, the reaction was pulled off heat and allowed to cool to room temperature. The nanoparticles were isolated by precipitation with ethanol and centrifuged at 8700 rpm for 5 min. Then, the nanoparticles were resuspended in chloroform. This process was repeated two more times.

### Time Study with P­(OPh)_3_

Nickel­(II) stearate (Ni­(C_18_H_37_CO_2_)_2_, 0.5 mmol), P­(OPh)_3_ (1 mmol), and 1-octadecene (ODE, 10 mL) were added to a 25 mL three-neck round-bottom flask. *N*,*N*-Diethylthiourea (3 mmol) and ODE (5 mL) were added to an attached pressure equilibrized addition funnel fitted with a thermocouple. The apparatus was placed under vacuum, while the round bottom was heated to 100 °C and held for 30 min. After refilling with nitrogen, the round bottom was heated to 195 °C, and then the addition funnel was heated to 195 °C with a heat gun. Once at temperature, the contents of the addition funnel were swiftly released into the reaction flask. The reaction mixture was held at a constant temperature and continuously stirred for 1, 5, 10, or 60 min. Then, the reaction was pulled off heat and cooled to room temperature. The nanoparticles were isolated by precipitation with ethanol and centrifuged at 8700 rpm for 5 min. Then, the nanoparticles were resuspended in chloroform. This process was repeated two more times.

### Computational Methods

Density functional theory (DFT) calculations were performed using the Vienna *Ab initio* Simulation Package (VASP).^39,40^ The Perdew–Burke–Ernzerhof (PBE) functional of generalized-gradient approximation (GGA) was employed for electron exchange and correlation.^41^ The electron–core interaction was modeled using the projector-augmented wave method (PAW).^42,43^ The van der Waals interaction was included by using the zero damping DFT-D3 method of Grimme *et al*.^44^ A kinetic energy cutoff of 500 eV was used for the plane-wave basis set. The Ni-terminated heazlewoodite (011̅), millerite (300), and α-NiS (102) facets were chosen to compute the adsorption energies of phosphine ligands because they were preferentially exposed based on their peak heights in the XRD patterns and nondistinct shape in the TEM. Slab models comprising 3 Ni–S layers and 18 surface nickel atoms with a vacuum layer of 15 Å were used to describe these facets, with the bottom Ni–S layer fixed during geometry optimizations. The Brillouin zones of the periodic slabs were sampled using a Monkhorst–Pack k-mesh of a (2 × 2 × 1). Adsorption energies (*E*_ads_) were calculated using the equation *E*_ads_ = *E*_ligand‑on‑surface_ – (*E*_surface_ + *E*_ligand_), where *E*_ligand‑on‑surface_, *E*_surface_, and *E*_ligand_ are the energies of the ligand-adsorbed-on-surface system, the clean surface, and the isolated ligand, respectively, so a negative *E*_ads_ indicates favorable binding.

### Characterization

Nanoparticles were characterized with powder X-ray diffraction (pXRD) using a Rigaku Smart Lab diffractometer with a Cu Kα X-ray (λ = 0.154 nm) radiation source set to 40 kV and 44 mA and a D/teX Ultra 250 1D silicon strip detector. pXRD patterns were acquired using a step size of 0.1° at a rate of 10° 2θ/min. Before analyzing the sample, it was dissolved in chloroform and drop-cast on a low-background pXRD plate. The patterns were matched to the corresponding phase using the ICSD database. Optical spectroscopy data were obtained using a Jasco V-670 Spectrophotometer and 1 cm path length quartz cuvettes. Heated spectrophotometry was conducted with a Mettler Toledo Spectrophotometer UV5 equipped with a CuveT thermal control unit and 1 cm path length quartz cuvettes. Spectra were taken at 85 °C. Transmission electron microscopy (TEM) images and energy-dispersive spectroscopy (EDS) were acquired on an FEI Tecnai Osiris TEM operated at 200 keV.

^31^P­{^1^H} NMR spectra were recorded on a 400 MHz Bruker console equipped with an 9.4 T Oxford Magnet and a 5 mm Z-gradient broadband probe. The samples were diluted with 600 μL of chloroform-*d* or benzene-*d*_6_, denoted with the spectra. All spectra were recorded at 25 °C.

## Results and Discussion

The impact coordinating ligands have on metal precursors to nanocrystal synthesis was first studied through the addition of three different phosphine ligands: PEtPh_2_, dppp, and xantphos. These ligands were added to nickel­(II) stearate in 1-octadecene before heating. Upon addition of the phosphines and heating to approximately 120 °C, the light blue-green nickel­(II) stearate changed color to brown-green solutions and, in the case of dppp, brown-orange by 100 °C.

Addition of *N*,*N*-diethylthiourea to the nickel­(II) containing solutions at 195 °C caused an immediate color change to black indicating the formation of nickel sulfide nanoparticles, which were allowed to grow for 1 h (Scheme 1). Without additional phosphine, the α-NiS (CN6) forms, but the addition of phosphine caused the formation of other phases such as millerite (CN5), heazlewoodite (CN4), and godlevskite (CN5/4). What role is the phosphine playing in phase determination?

**Scheme 1:**
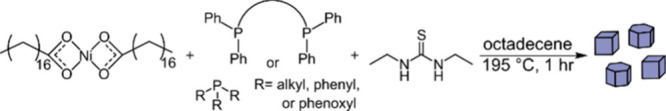
General Reaction Scheme

These nickel sulfides have various oxidation states of nickel (Ni^+^, Ni^2+^, Ni^3+^) despite the starting material being Ni^2+^. We envision that the 6 equiv. of sulfur precursor provides a redox sink, for whatever phase is favored, since sulfur can form S_n_^2–^ species in solution.

Nanocrystal nucleation pathways are notoriously difficult to study. The high temperatures can cause nonintuitive pathways, and there are highly complex reaction mixtures with multiple roles for ligands and solvents. We are rarely sure of molecular decomposition mechanisms, and the products, unless clusters are formed, are inherently inhomogeneous with millions of possible particle structures. To study these complex systems, we make a number of assumptions. By choosing the same reagents with the same temperatures, we assume that the molecular mechanisms that preclude nanocrystal formation are identical.

### Bite Angle

NiL_2_P_2_ molecular structures can show both tetrahedral and square planar coordination, and the bite angles of the bidentate ligand can influence molecular geometry. One might imagine a nickel atom approaching the surface of a growing nanoparticle ligated by a bidentate phosphine that might influence whether it enters a tetrahedral (angles of 109.5°) or octahedral (angles of 90°) site. For this reason, we tested the roles of dppp (bite angle 91°) and xantphos (bite angle 108°) to see if perhaps the former preferred structures with octahedral coordination and the latter those with tetrahedral coordination.^37^

Both dppp and xantphos showed similar behavior in their influence of phase in the formation of nickel sulfides, and their bite angles showed no trend in coordination (Figure 2). Without phosphine, the chosen reaction conditions (195 °C, 1 h, Ni:S 1:6) gave α-NiS (CN6). When dppp was added, millerite (CN5) formed at Ni:P ratios of 1:1 and 1:2; heazlewoodite (CN4) formed at a ratio of 1:4. It is notable that added dppp in high ratios gave tetrahedral coordination despite having a bite angle of only 91°. For xantphos, millerite (CN5) resulted from all ratios of Ni:P tested. Xantphos did not favor the tetrahedral coordination as hypothesized, despite having a bite angle of 108°.

**Figure 2:**
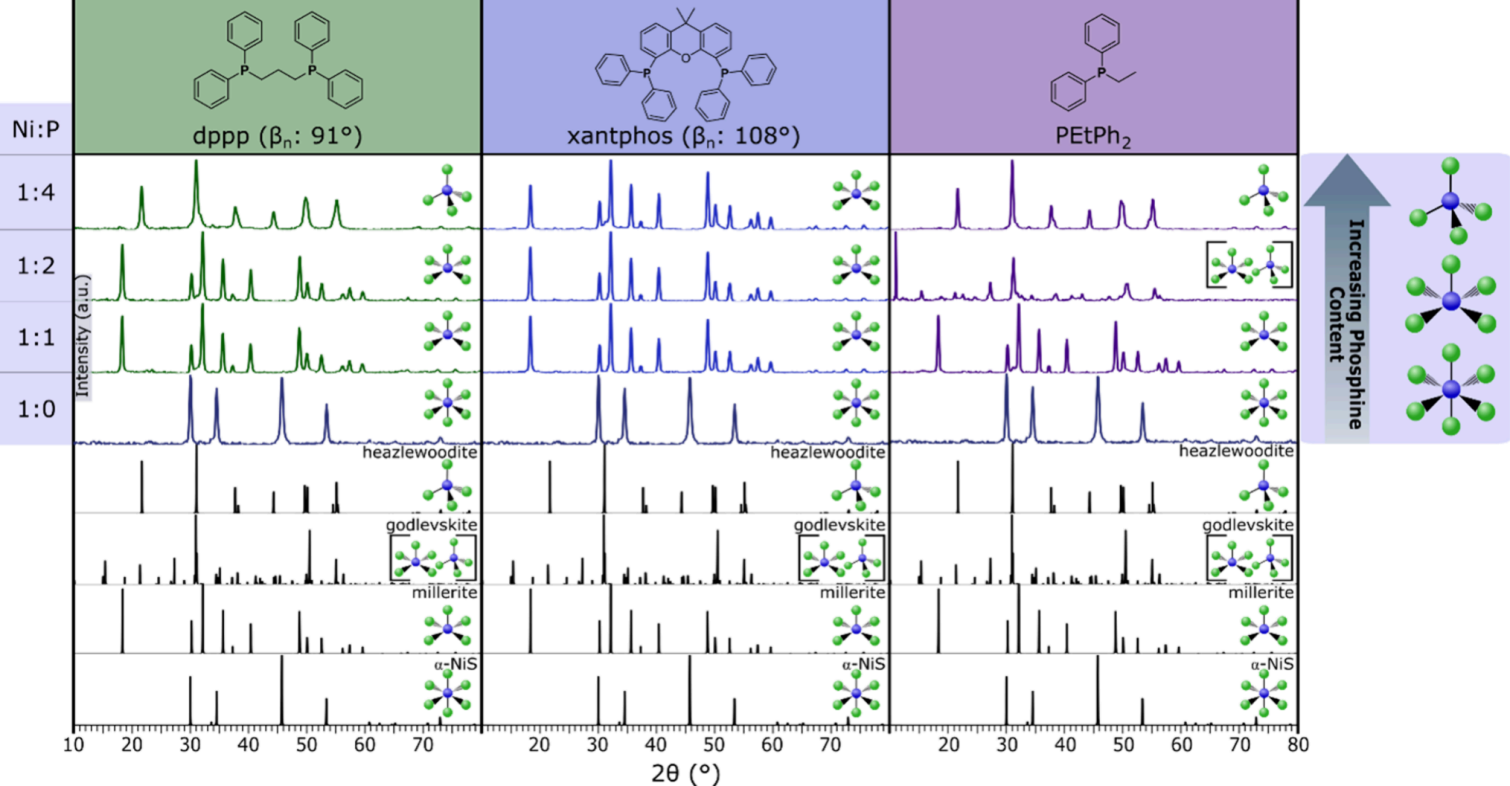
pXRD patterns for the products of the reaction of nickel­(II) stearate and *N*,*N*-diethylthiourea at 195 °C in the presence of dppp (left), xantphos (center), and PEtPh_2_ (right) with varying Ni:P ratios of 1:4, 1:2, 1:1, and 1:0. Increasing the phosphine concentration in solution decreased the coordination number of the nickel in the product nickel sulfide phase. Bite angles for the bidentate ligands are after their abbreviations. To denote the phase produced, coordination figures are to the right of each pattern. The tetrahedral CN denotes heazlewoodite (CN4), the square pyramidal CN denotes millerite (CN5), the tetrahedral and square pyramid CNs in brackets denote godlevskite (CN5/4), and the octahedral CN denotes α-NiS (CN6) (α-NiS: 1010435 COD; millerite: 9004078 COD; godlevskite: 9013880 COD; heazlewoodite: 36338 ICSD).

As further evidence that the bite angle of bidentate ligands did not have an effect on the phase of nickel sulfide that formed, PEtPh_2_ was employed, as it has a similar electron donation ability to dppp. The addition of PEtPh_2_ caused the formation of millerite (CN5) at 1:1, godlevskite (CN5/4) at 1:2, and heazlewoodite (CN4) at 1:4 (Figure 2, TEM images of 1:4 Figure S5). This trend is almost identical to that of its bidentate cousin, dppp, and thus the bite angle of bidentate ligands is not a good explanation for the observed trends in phase.

### Coordination Site Blocking

For the three phosphines studied above, in all cases, the addition of phosphine caused a lower coordination number around nickel in the resulting nickel sulfide compared to the control without phosphine. An explanation for this trend is that the phosphine blocks coordination sites during incorporation into the growing nickel sulfide.

As a test, the sulfur concentration was varied in reactions with 1 mol equivalent of each phosphine to observe competition between the sulfur and phosphine for coordination sites on the nickel. All three phosphines again showed similar trends (Figure 3). At low sulfur concentrations (Ni:S 1:1), heazlewoodite (CN4) formed for xantphos and PEtPh_2_. Under these conditions, nickel did not react when coordinated with dppp. While a crystalline product was formed, the pattern matched a control reaction of nickel­(II) stearate and dppp (Figure S2). We assume this to be a nickel­(II) stearate-dppp complex. With the addition of dppp and Ni:S 1:2, heazlewoodite (CN4) forms, and godlevskite (CN5/4) forms for xantphos and PEtPh_2_. At Ni:S 1:4, godlevskite (CN5/4) forms with dppp and xantphos, and a mixture of godlevskite (CN5/4) and millerite (CN5) is produced with PEtPh_2_. Millerite (CN5) forms for all three phosphines at Ni:S 1:6. In all cases, increasing the sulfur ratio increases the coordination number in the product nickel sulfide, and we interpret this to mean that the thiourea can compete with the phosphine for coordination to the nickel centers at high concentrations to achieve up to CN5.

**Figure 3:**
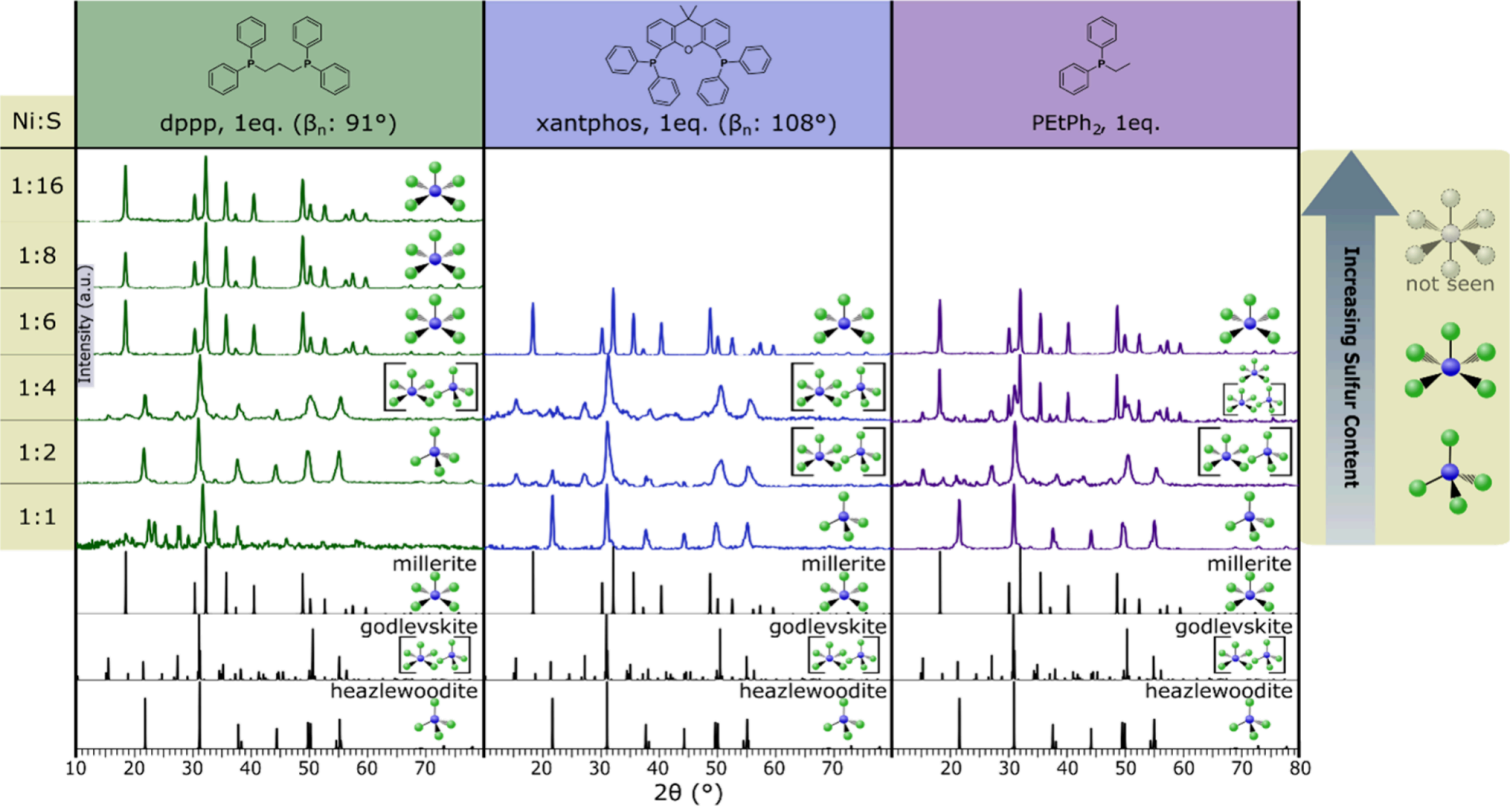
pXRD patterns for the products of the reaction of nickel­(II) stearate and *N*,*N*-diethylthiourea at 195 °C in the presence of dppp (left), xantphos (center), and PEtPh_2_ (right) with varying Ni:S ratios. Increasing the sulfur concentration in solution increases the coordination number of the nickel in the product nickel sulfide phase. Interestingly, even at high sulfur concentrations (1:16), CN6 is not seen. At Ni:S 1:1 in the presence of dppp, an assumed nickel­(II) stearate and dppp complex form (Figure S2). To denote the phase produced, coordination figures are to the right of each pattern. The tetrahedral CN denotes heazlewoodite (CN4), the square pyramidal CN denotes millerite (CN5), and the tetrahedral and square pyramid CNs in brackets denote godlevskite (CN5/4) (millerite: 9004078 COD; godlevskite: 9013880 COD; heazlewoodite: 36338 ISCD).

Intriguingly, it appears that even at Ni:S 1:8 and 1:16 the *N*,*N*-diethylthiourea cannot outcompete the phosphines completely. Even at these extremes, CN6 nickel products were not observed. Without phosphine present, these conditions give α-NiS (CN6). This implies that the phosphine is blocking sulfur coordination to at least one equivalent.

Throughout this discussion, it is assumed that the phosphine is not creating phosphine-sulfides as an intermediate. To test the assumption, an NMR scale experiment was performed of *N*,*N*-diethylthiourea and dppp in an ODE, heated to 195 °C for 1 h. The resulting ^31^P NMR has a shift at −16.2 ppm in agreement with dppp (Figure S3). A small peak at 43.1 ppm is apparent, which aligns with a standard of dppp sulfide. Because the dppp sulfide peak is miniscule in comparison to the free dppp comprising likely far less than 1%, the phosphine sequestering sulfur from the thiourea quickly after injection in the syntheses to nickel sulfides was ruled out.

### Electronics vs Sterics

The rate at which phosphines are displaced on metal centers is controlled by steric parameters (cone angle), electronic parameters (electron donation), or some blend of the two. If the electron donation abilities of the phosphines are most important to phase control, then weakly bound phosphines should be more easily displaced by *N*,*N*-diethylthiourea and would form products with a high CN of sulfurs. Contrarily, strongly electron donating phosphines would form low CN phases. Alternatively, if the steric is the more important factor to phase control, one might predict that large ligands on a *d* ^8^ four-coordinate nickel center would inhibit associative coordination of thiourea and yield products with low CNs of sulfurs; small ligands would produce products with high CNs of sulfur. Therefore, studying the trends in electronic parameters and cone angles of a series of phosphine ligands will reveal the mechanism behind phase control.

To test whether sterics or electronics of the phosphine are the more important factor to phase control, a collection of monodentate phosphine ligands—PMe_2_Ph, P­(OPh)_3_, PCy_3_, PBu_3_, TOP, and P­(*p*-tol)_3_—was explored. These phosphines cover a range of Tolman electronic parameters and cone angles (Table S1 and Figure 4).

**Figure 4:**
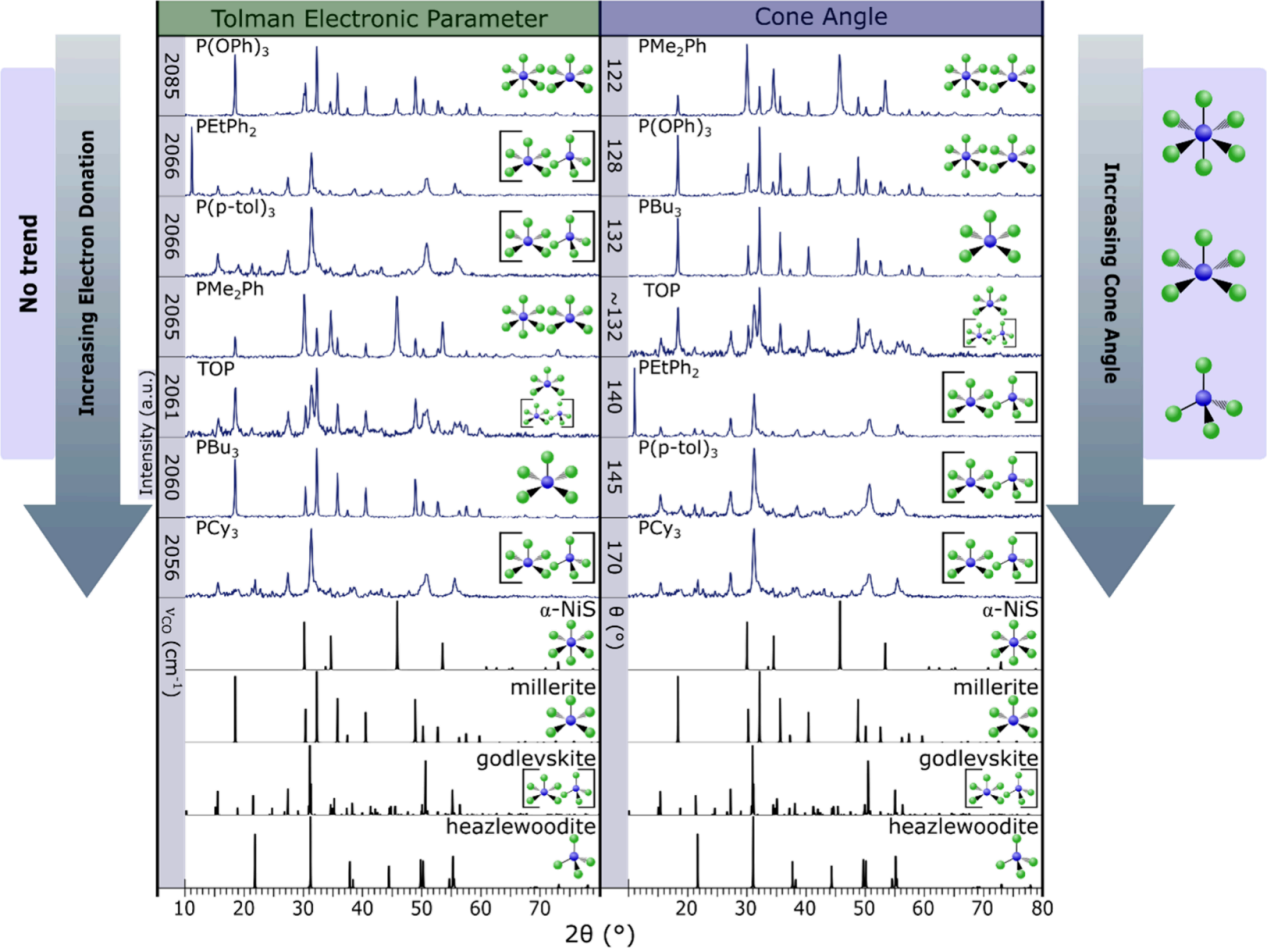
pXRD patterns for the products of the reaction of nickel­(II) stearate and *N*,*N*-diethylthiourea at 195 °C in the presence of varying monodentate phosphines—PMe_2_Ph, P­(OPh)_3_, PBu_3_, TOP, PEtPh_2_, P­(*p*-tol)_3_, and PCy_3_, organized in two different ways. When ordered via the phosphine electron donation ability (left), no trend is seen, but when ordered via cone angle (right), a trend can be seen, increasing the cone angle decreases the coordination number of the nickel sulfide produced. To denote the phase produced, coordination figures are to the right of each pattern. The square pyramidal CN denotes millerite (CN5), the tetrahedral and square pyramid CNs in brackets denote godlevskite (CN5/4), and the octahedral CN denotes α-NiS (CN6) (α-NiS: 1010435 COD; millerite: 9004078 COD; godlevskite: 9013880 COD; heazlewoodite: 36338 ISCD).

When 1 equiv. of each phosphine was added, the major product was millerite (CN5), except for the smallest and least electron donating phosphine, P­(OPh)_3_, which formed a mixture of millerite (CN5) and α-NiS (CN6) (Figure S4).

When 2 equiv. of each monodentate phosphine were added, the product phases varied among α-NiS (CN6), millerite (CN5), and godlevskite (CN 5/4). When the results were organized by their Tolman electronic parameters, no trend was easily identified (Figure 4). It appears that the electron donation abilities of phosphines do not have a large effect on the coordination number of the nickel in the product nickel sulfides.

When the results instead were organized by the cone angle of the added phosphines (Figure 4), a trend was readily seen. Increasing the cone angle of the added phosphine decreased the coordination number around the nickel in the resulting phase. The smallest cone angle, PMe_2_Ph, produced millerite (CN5) and α-NiS (CN6). As the cone angle increased, more millerite (CN5) formed with P­(OPh)_3_ addition and all millerite (CN5) with the addition of PBu_3_. A mixture of godlevskite (CN5/4) and millerite (CN5) forms with TOP, and godlevskite (CN5/4) forms with PEtPh_2_, P­(*p*-tol)_3_, and PCy_3_. It can be concluded that the cone angle is the more important factor in how phosphines dictate the phase of the resultant nickel sulfides and indicates an associative molecular mechanism.

TEM images shown in Figure S5 were taken of the phase-pure samples—millerite (CN5; PBu_3_, 2 equiv.) and godlevskite (CN5/4; PCy_3_, 2 equiv.)—from this study, along with pure heazlewoodite (CN4; PEtPh_2_, 4 equiv.) and pure α-NiS (CN6; no phosphine) from Figure 2. All phases exhibit nondiscrete shapes and have a tendency to stack. Mixed phases from this study—2 equiv. additions of P­(OPh)_3_ (α-NiS (CN6) and millerite (CN5), Figure S10) and TOP (millerite (CN5) and godlevskite (CN5/4), Figure S12)—were also imaged. Neither mixture had obvious differences in morphology, but variation in size can be seen in the mixed phase sample from the addition of P­(OPh)_3_ (Figure S10). Because of stacking in all of the samples, phase characterization from TEM is convoluted.

The series of phosphine ligands chosen for study was limited at both the largest and smallest sizes. Bulky phosphine ligands can have reactive sites far from the P center on the substituents. Syntheses of nickel sulfides were attempted with P­(*o*-tol)_3_ (192°), but unanticipated cyclometalation^45–47^ caused different reactivity of the active metal precursor in solution and a deviation from the trend, forming α-NiS (CN6) and millerite (CN5) (Figure S15). Other bulky phosphines with similar placements of active groups should be used with caution. As such, the extremes of bulky phosphines that can be used in this study are limited. The employment of smaller phosphines is also limited, but instead by having low boiling points incompatible with the high temperatures of nanocrystal synthesis. The smallest phosphine used in this study, PMe_2_Ph, has a boiling point at the standard reaction temperature (195 °C), so a temperature of 190 °C was used instead.

### Molecular Effects

The effect of the phosphine cone angle on the phase of the product nickel sulfides may have its origin in molecular processes that prelude nanocrystal formation. When phosphines are added to the nickel­(II) stearate solution at temperatures above 100 °C, a color change from blue-green to orange or green was observed consistently, suggesting the displacement of some of the coordination sites around the nickel complex with phosphine. UV–vis of a solution of dppp and nickel stearate showed an absorption maxima around 380 nm. The extinction coefficient of ε = 99 cm^–1^ M^–1^ suggests a noncentrosymmetric d-d transition, consistent with a tetrahedral complex (Figure S17). Phosphines are indeed interacting with the nickel center before nanocrystal formation and may play a role in *N*,*N*-diethylthiourea attachment and decomposition, leading to phase control.

EDS (Figures S6–S9, S11, and S13) of the particles shows consistent inclusion of phosphorus, which indicates surface phosphine coordination. We make the assumption that the totality of the coordinating phosphines is only replaced once the nickel is part of the crystal.

Under a four-coordinate nickel complex, bulky phosphines would slow associative mechanisms for *N*,*N*-diethylthiourea coordination, leading to low coordination number species, as was observed experimentally. Phosphines may therefore influence the phase at the earliest nucleation steps.

Clues to the nucleation behavior can be deduced from the growth of the two different phases with time. A standard reaction of nickel­(II) stearate and P­(OPh)_3_ (Ni:P 1:2) with *N*,*N*-diethylthiourea, which was previously shown to produce millerite (CN5) and α-NiS (CN6) at 1 h, was instead stopped at 1, 5, 10, and 60 min (Figure 5). At 1 min, α-NiS (CN6) was the primary product with a small amount of millerite (CN5). As the reaction progressed, millerite (CN5) becomes increasingly dominant with α-NiS (CN6) becoming the minority phase (Table S3).

**Figure 5:**
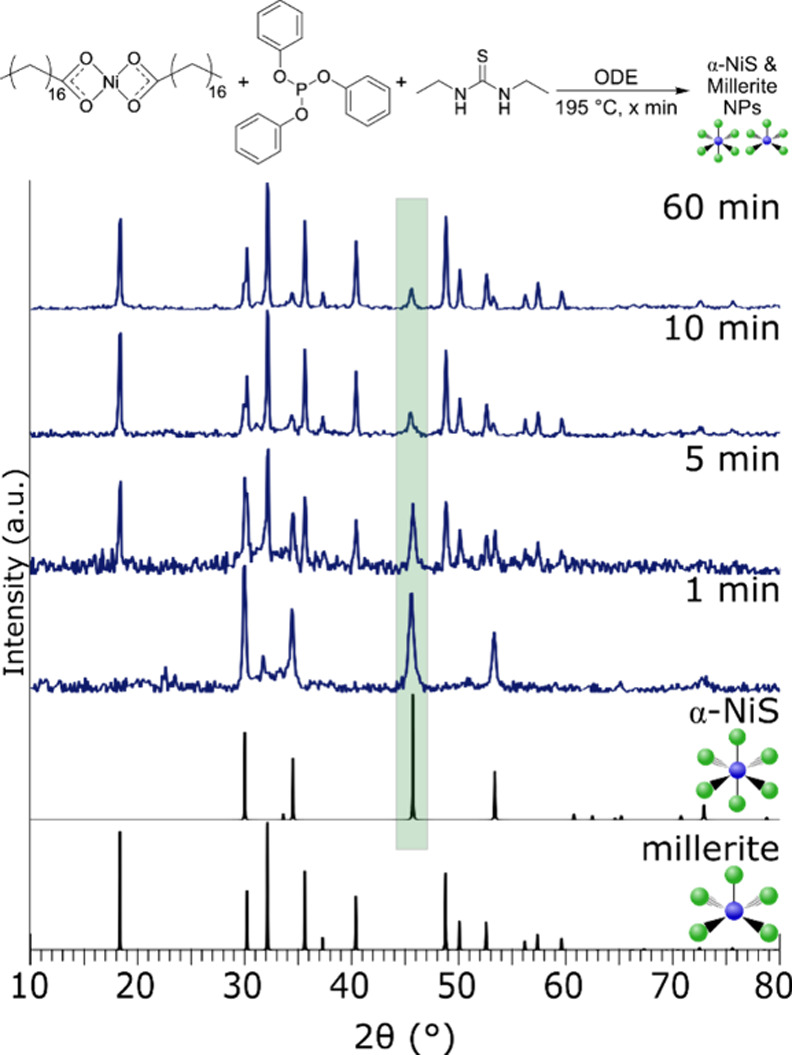
(Top) Reaction scheme for time studies with addition of 1 mmol of P­(OPh)_3_. (Bottom) pXRD patterns of products from 1, 5, 10, and 60 min reactions. As reaction time increases, millerite (CN5) becomes the major product instead of α-NiS (CN6). Green box highlighting the main α-NiS (CN6) peak for visibility (α-NiS: 1010435 COD; millerite: 9004078 COD).

As well as becoming the majority product, millerite (CN5) crystallites grow in size through the time frame of the reaction. Only one distinct reflection at 32° 2θ is present at 1 min with low intensity; therefore, size cannot be calculated. Via the Halder–Wagner method, at 5 min, millerite (CN5) was calculated to be 13.9 ± 0.4 nm, and at 10 min, the crystallite size exceeds 60 nm where diffraction-based sizing of crystallites becomes only qualitative.^48^

α-NiS (CN6) crystallites, by contrast, grow in the first five min, but then, the size becomes stable. Line broadening of the reflection at ∼45.7° 2θ, which is unique to α-NiS (CN6), (Figure S19) indicates that between 1 and 5 min there is a small amount of growth, but at times beyond 5 min, the peak width remains the same with crystallite sizes of ∼20–22 nm. Since the α-NiS (CN6) crystallites are not shrinking as the millerite (CN5) grows at the longer reaction times, we can infer that millerite (CN5) nucleates independently of α-NiS (CN6) and does not grow at the expense of the α-NiS (CN6) in a ripening process. Evidence for separate growth can be seen in TEM images of the 1 h sample (Figure S10), where two different sizes of crystals can be seen.

Since we know that a phosphine is needed in this reaction to obtain millerite (CN5) (beyond only α-NiS (CN6)), and millerite (CN5) is nucleating separately from α-NiS (CN6), it can be inferred that the phosphines are influencing phase during nucleation, rather than solely on the full nanoparticle surface. We can also infer from this information that there is not a common nucleated phase that then differentiates.

### Surface Effects

Phosphine cone angle likely influences phase control through interactions on nanocrystal surfaces, in addition to the nucleation behavior. Indeed, a nanocrystal surface provides far more steric bulk than the three other coordination sites on a tetrahedral nickel complex. Nickel coordinated by bulky phosphines would be challenged to nestle into high-coordination sites on the nanocrystal surface and instead would be restricted to low coordination surface sites. Surface ligand-induced bulk phase transformation has been reported before in other colloidal nanoparticle systems such as Cu, Pd, and CdSe@CdS.^49–51^

To better understand the surface mechanism, the adsorption of phosphine ligands with varying cone angles on nickel sulfide surfaces was examined through density functional theory (DFT) calculations. Three surfaces with three different types of coordination were chosen: heazlewoodite (CN4) (011̅), millerite (CN5) (300), and α-NiS (CN6) (102); since the particles by TEM had nondistinct shapes, the surfaces chosen for modeling were from the strongest XRD reflections. Various adsorption configurations of two phosphine ligands PMe_2_Ph and PEtPh_2_ with small and large cone angles (122 and 140°, respectively) on the surfaces were then explored by DFT geometry optimization. The computed average adsorption energies (Table 1) show that the bulkier PEtPh_2_ ligand has stronger adsorption on both heazlewoodite (CN4) and millerite (CN5), while both ligands have stronger binding on heazlewoodite (CN4) than on millerite (CN5). As shown in Figure S21, on the heazlewoodite (CN4) surface, one of the phenyl groups of PEtPh_2_ ligand is in close contact to the surface, while the phenyl group of PMe_2_Ph is farther away from the surface, as is allowed by the small cone angle. As a result, the strong adsorption of the ligand with a high cone angle partially originates from the dispersion interaction between pendant phenyl groups and the surface.

**Table 1:** DFT-Computed Average Adsorption Energies of PEtPh_2_ and PMe_2_Ph on Heazlewoodite and Millerite Surfaces

phosphine	heazlewoodite (CN4)	millerite (CN5)
PEtPh_2_	–3.92 eV	–2.30 eV
PMe_2_Ph	–3.05 eV	–2.19 eV

That is not to say that steric effects are unimportant; the binding of small and large phosphines is dependent on the type surface presented, with lower coordination number surface nickel seeing the largest effect. The adsorption energy difference between the small and large cone angle ligands in the case of heazlewoodite (CN4) is approximately 0.90 eV, while the difference in the case of millerite (CN5) is much smaller at approximately 0.11 eV. Due to the higher coordination number, nickel atoms on the surface of millerite (CN5) are more deeply situated, and steric hindrance of pendant phenyl groups of PEtPh_2_ tempers the advantage of added dispersion forces (Figure S21).

The DFT calculations are consistent with the experimental observations. The addition of 2 equiv. of bulky PEtPh_2_ forms the CN5/4 product because higher dispersion forces between the ligand pendant groups and the surface, as well as lower surface steric hindrance, favor surface nickel atoms with low coordination. In contrast, adding 2 equiv. of the small PMe_2_Ph forms high-coordination CN6 and CN5 products. In this case, steric hindrance and dispersion forces are lessened, and the surface nickel atom is better satisfied by high coordination to sulfur species in the nanocrystal than with ligand (Figure 4).

The calculated adsorption energies of the phosphines on α-NiS (CN6) are not directly comparable to those of the other two phases, as major surface reconstructions occurred upon ligand addition after geometry optimizations (Figure 6). When PEtPh_2_ was added to the surface of α-NiS (CN6) (at a density of 1P:16Ni), three nickel centers changed from CN5 to CN4, while two changed from CN5 to CN3. Multiple Ni–S bonds were broken: two in the first coordination sphere, two in the second, and two in the third. When PMe_2_Ph was added, a similar rearrangement can be seen, where three nickel centers changed from CN5 to CN4, and two centers changed from CN5 to CN3 (Figure 6), even more Ni–S bonds were broken: two in the first coordination sphere, three in the second, and two in the third. The DFT calculations suggest that phosphine ligands, regardless of cone angle, highly destabilize the CN5 α-NiS (CN6) surface, which agrees with the experimental observation that no pure α-NiS (CN6) was produced in the presence of any of the phosphine ligands (Figure 2).

**Figure 6:**
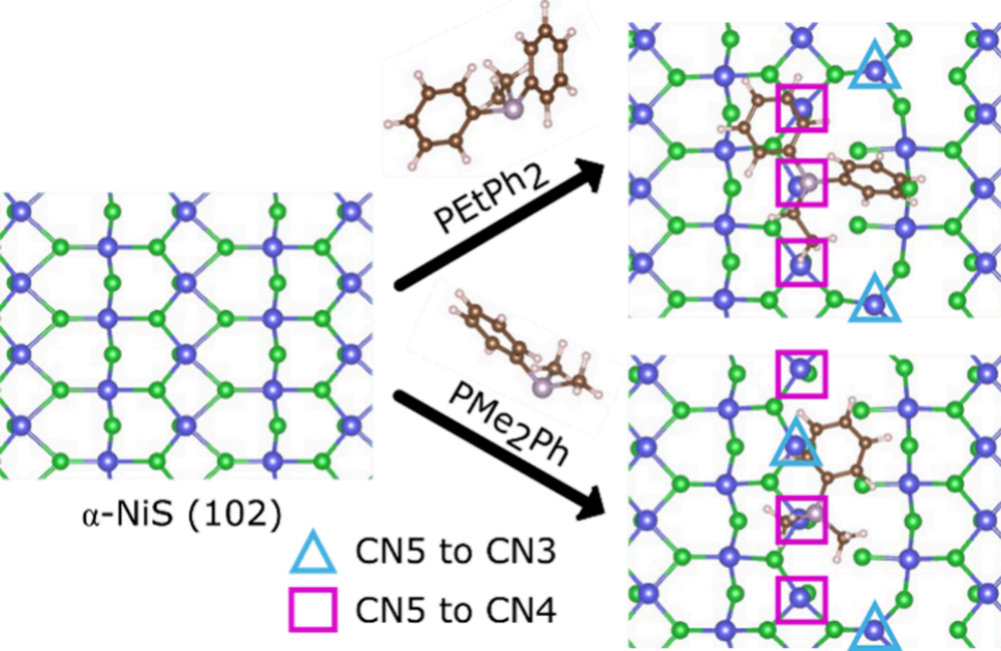
Adsorption-induced structural changes from DFT geometry optimization of phosphines (PEtPh_2_ or PMe_2_Ph) on α-NiS (102). The change from CN5 to CN4 is denoted by pink squares, and the change from CN5 to CN3 is denoted by blue triangles.

The DFT calculations in Figure 6 suggest surface reconstructions of α-NiS (CN6) by phosphine ligands, but whether the effect can affect the crystalline phase beyond the surface remains to be seen. To test this experimentally, batches of α-NiS (CN6) particles (22–24 nm, with standard deviations of ∼3.4 nm) and a monodentate phosphine (PEtPh_2_ or P­(OPh)_3_, 1 mmol) were heated to 195 °C in ODE for 1 h (Figure 7). When the larger PEtPh_2_ was used, the resulting nanoparticle showed a change in phase from α-NiS (CN6) to heazlewoodite (CN4). So in conjunction with the DFT calculations, it can be concluded that phosphines destabilize α-NiS (CN6) surfaces, and the surface chemistry triggers phase changes throughout the crystal to lower coordination number structures, with bulky ligands causing the more drastic change.

**Figure 7:**
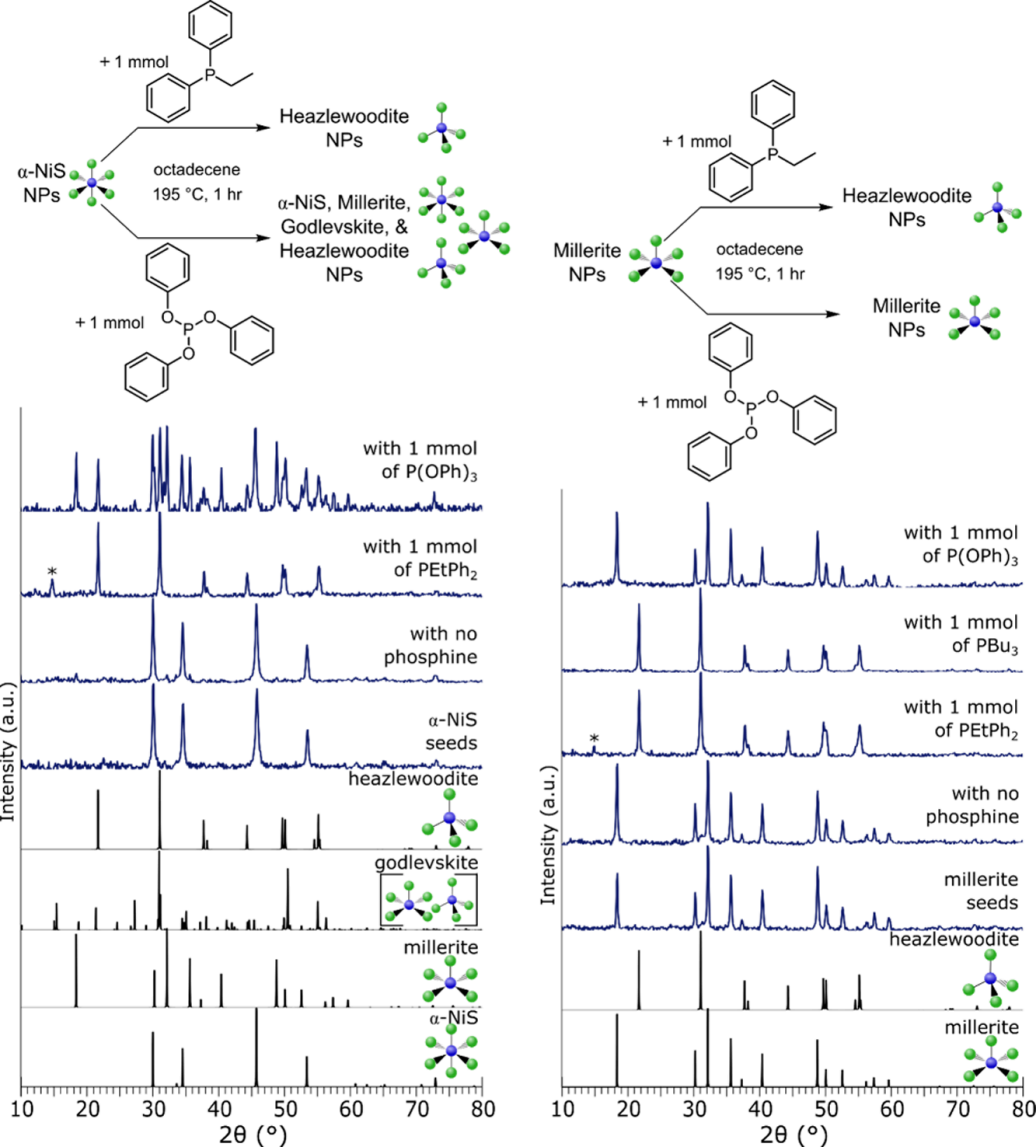
(Left top) Reaction scheme for reactions of α-NiS combined with PEtPh_2_ or P­(OPh)_3_. (Left bottom) pXRD patterns for the reaction products with PEtPh_2_, P­(OPh)_3_, and no phosphine. PEtPh_2_ addition caused a transformation from CN6 to CN4, while P­(OPh)_3_ addition caused a transformation from CN6 to a mixture of CN6, 5, and 4 products. No change is seen when α-NiS (CN6) is heated alone. (Right top) Reaction scheme for reactions of millerite (CN5) combined with PEtPh_2_ or P­(OPh)_3_. (Right bottom) pXRD patterns for the reaction products with PEtPh_2_, PBu_3_, P­(OPh)_3_, and with no phosphine. PEtPh_2_ and PBu_3_ addition caused a transformation from CN6 to CN4, while P­(OPh)_3_ addition caused no transformation. No change is seen when millerite (CN5) is heated alone. The * marks a peak that is assigned to a repeating defect or vacancy within the crystal structure (α-NiS: 1010435 COD; millerite: 9004078 COD; godlevskite: 9013880 COD; heazlewoodite: 36338 ISCD).

Added phosphines can also destabilize millerite (CN5) surfaces and induce phase changes. Millerite (CN5) particles (10–15 nm, with batch deviations of ∼1.4 nm) and a monodentate phosphine (PEtPh_2_, P­(OPh)_3_, PBu_3_, 1 mmol) were heated to 195 °C in ODE for 1 h (Figure 7). When the large PEtPh_2_ was used, the resulting nanoparticle showed a change in phase from millerite (CN5) to heazlewoodite (CN4). The same transition also occurred for PBu_3_, which has a smaller cone angle but a similar electronic donation ability to PEtPh_2_. In contrast, when the smaller and weaker binding P­(OPh)_3_ was added, no change in the phase was seen. These experiments show that phosphines can also destabilize millerite (CN5) surfaces, but the effect is smaller than that with α-NiS (CN6), since P­(OPh)_3_ had no effect.

The phase transitions of preformed nickel sulfide particles by phosphines to sulfur poor phases could have an alternative explanation. Each of the transitions noted requires removal of S from the crystals, presumably to a phosphine-sulfide (R_3_P:S) complex. It has been previously observed that phosphines can induce phase transitions in iron and copper sulfides and nickel, tin, and cobalt selenides to chalcogen poor phases.^13,52,53^ The amount of extraction and the extent of the phase transition could be driven by the thermodynamic stability of the phosphine-sulfide complex and therefore correlate with the phosphine electronic parameter.

It is likely that both the stability of the phosphine-sulfide molecular complex and the surface effects are at play under monomer-depleted and ligand-rich conditions. Such a hypothesis has not been rigorously tested in any of the examples above, and this is a potential avenue for future research. We do know, however, that during syntheses under reagent-rich conditions, the cone angle effect outweighs the electronic effects, as shown above.

## Conclusions

To synthesize specific materials for desired applications, we need a chemical toolbox that allows us to select specific phases of metal chalcogenides. While the toolbox is growing, some phases still cannot be made pure. Bottom-up phase control studies thus far focus mainly on accessing phase-pure products, through changing the reactivity of the chalcogenide precursor^2–5^ as well as the counterion on the metal precursor counterion.^7,8^ While cation exchange studies focus on the metal, this method comes with limitations, as some metal exchanges can cause changes to the crystal structure^15,16^ or morphology.^18^ Ideally both phase purity and structural control can be achieved in a single synthesis. Here, for the first time, we focus on utilizing coordination chemistry concepts to access phase-pure products while controlling the resulting crystal structures. In summary, we were able to influence nickel sulfide formation between four pure phases—α-NiS (CN6), millerite (CN5), godlevskite (CN5/4), and heazlewoodite (CN4)—by the addition of a phosphine to a synthesis.

Phosphines induce nickel to take on lower coordination sites in the product phase. DFT calculations and experiments show that bulkier phosphines have the largest effect, which can be ascribed to both phase selection at the nucleation stage and surface reconstruction of the crystallites.

Phosphines bind to the molecular nickel species before nanocrystal formation, forming tetrahedral complexes. The bulk of the phosphine likely influences the rate of associative addition of the sulfur precursor, *N*,*N*-diethylthiourea. Experimental evidence shows that the phosphine influences which phase nucleates in the cases of α-NiS (CN6) and millerite (CN5).

It is possible that in applying the concept of steric bulk to influence nucleation behavior to other metals, bulky phosphines may have the opposite effect. If the intermediate metal complex is six-coordinate and must go through a dissociative mechanism, then bulky phosphines will likely speed up the reaction.

Through a combination of DFT calculations and experimental syntheses, we also found evidence that phosphines cause surface reconstructions to low coordination number nickel sites that induce a bulk phase change. α-NiS (CN6) seeds can be turned into heazlewoodite (CN4), godlevskite (CN5/4), and millerite (CN5) by the addition of a phosphine. Similarly, millerite (CN5) can be turned into heazlewoodite (CN4). This work shows that bulky phosphines had a larger effect because of their steric crowding at the surface and they also bind more strongly to the surface because of dispersion effects.

Cation coordination number in crystalline materials can be influenced by the host material in cation-exchange processes;^10^ however, to our knowledge, this is the first time that cation coordination has been a rational target for influencing phase control in bottom-up colloidal synthesis, including the nucleation step. Such an approach may find future use not only in the synthesis of binary materials but also in tricky ternary materials, where two cations must be cajoled into differing crystallographic sites.

## Supplementary Material



## Notes

Powder X-ray diffraction patterns, pXRD refinement values and residuals, TEM images, optical spectroscopy spectra, nuclear magnetic resonance spectra, monodentate phosphine parameters, and DFT models (PDF)

## Abbreviations

hcp: hexagonal closed packed
ccp: cubic closed packed
CN: coordination number
ODE: 1-octadecene
PEtPh2: ethyldiphenylphosphine
P­(OPh)­3: triphenylphosphite
PCy3: tricyclohexylphosphine
PBu3: tributylphosphine
TOP: trioctylphosphine
P­(*p*-tol)­3: tri­(*p*-tolyl)­phosphine
(P­(*o*-tol)­3: tri­(*o*-tolyl)­phosphine
PMe2Ph: dimethylphenylphosphine
dppp: 1,3-bis­(diphenylphosphino)­propane
xantphos: 9,9-dimethyl-4,5-bis­(di-*tert*-butylphosphino)­xanthene
DFT: density functional theory
VASP: Vienna *Ab initio* Simulation Package
PBE: Perdew–Burke–Ernzerhof
GGA: generalized-gradient approximation
pXRD: powder X-ray diffraction
NMR: nuclear magnetic resonance
TEM: transmission electron microscope
EDS: electron-dispersive spectroscopy
