# Supplementary material for: Directing Cation Coordination and Phase in Nickel Sulfide Nanocrystals through the Addition of Phosphines
Source: Chem Mater. 2025 Dec 29;38(1):190–201. doi: 10.1021/acs.chemmater.5c02148 (PMC12805513; doi:10.1021/acs.chemmater.5c02148)
Supplement: Supplementary file 1 [file cm5c02148_si_001.pdf]

# Directing Cation Coordination and Phase in Nickel Sulfide Nanocrystals through the Addition of Phosphines

Emma J. Endres,[ab] Yiming Chen,[ac] De-en Jiang,[bc] and Janet E. Macdonald[ab*]

[a]Department of Chemistry, Vanderbilt University, Nashville, Tennessee 37235, United States

[b]Vanderbilt Institute for Nanoscale Science and Engineering, Nashville, Tennessee 37235, United States

[c]Department of Chemical and Biomolecular Engineering, Vanderbilt University, Nashville, Tennessee 37235, United States

*Email: janet.macdonald@vanderbilt.edu

**Table of Contents**

Figure S1: Reaction set up……………………………………………………………………2

Figure S2: Nickel(II) stearate and dppp control pXRD……….………………………….....2

Figure S3: Dppp sulfide control $^{31}$P NMR………..……………………..…………….…...3

Scheme S1: Molecular structures of phosphines…………………………………………….4

Table S1: Tolman electronic parameters and cone angles………………….……………….…...4

Figure S4: 1 equivalent monodentate addition pXRD.……………………………….……...5

Figure S5: TEM images of phase pure samples………………………………………………6

Figure S6-7: EDS spectra of phase pure α-NiS and millerite……………………………….....7

FigureS8-9: EDS spectra of phase pure godlevskite and heazlewoodite……………………...8

Figure S10: TEM images of mixed phase product from $P(OPh)_3$ synthesis………………………..9

Figure S11: EDS spectrum of the mixed phase product from $P(OPh)_3$ synthesis…………………9

Figure S12: TEM images of mixed phase product from TOP synthesis…………………………..10

Figure S13: EDS spectrum of the mixed phase product from TOP synthesis……………………..10

Table S2. Rietveld refinements of products produced with varying cone angles………………….11

Figure S14. Phase refinement for reactions with 2 equivalents of monodentate addition………...12

Figure S15: P(o-tol)$_3$ pXRD……………………………………………………..……........13

Figure S16: Ni:P 1:2 and 1:4 $^{31}$P NMR……………………...…………………….……...13

Figure S17: UV-vis and extinction coefficient of $Ni(dppp)_2$……………………………..……14

Table S3: Refinements for $P(OPh)_3$ time study…..…………………...…………………….…...15

Figure S18: Refinements for $P(OPh)_3$ time study…..…………………………………........15

Figure S19: α-NiS peak line broadening………………..………………..…………..….....….16

Figure S20: DFT clean surfaces without phosphine ………...………………….………………17

Figure S21: DFT-optimized adsorption structures and energies...………… ……………...…….18

Table S4. Summary table of syntheses that produce phase pure products………………………20

References………………………………………………………………………………..21

*Reaction set up:*

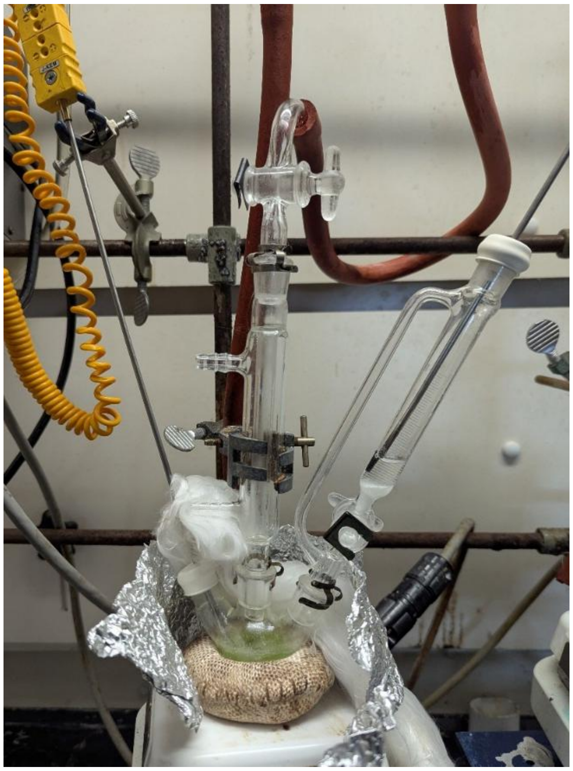

**Figure 1.** The reaction set up: a 25 mL three neck round bottom flask with a condenser and gas adapter attached to the neck and a pressure equilibrized addition funnel on one arm. The round bottom flask and addition funnel are both equipped with thermocouples.

*Nickel(II) stearate and dppp control:*

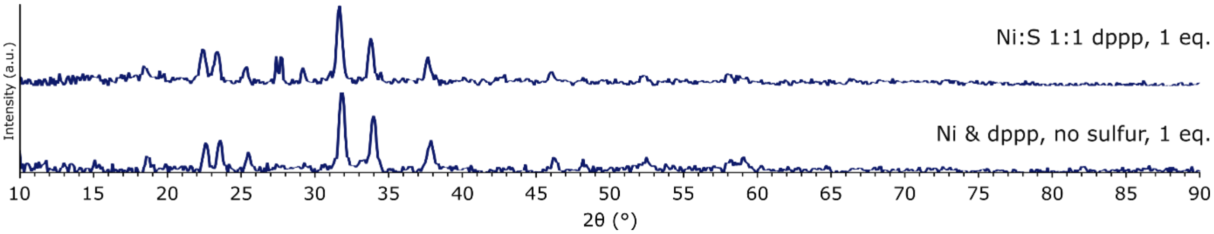


**Figure S2.** pXRD patterns for the products of the reaction of (top) nickel(II) stearate with one equivalent of dppp and 1:1 Ni:S and (bottom) a control reaction of nickel (II) stearate with one equivalent of dppp, no sulfur addition. As the two patterns are almost identical, it is assumed that no sulfur is present in the crystalline product from the top reaction (Ni:S 1:1) We assume this to be a nickel(II) stearate-dppp complex.

*Dppp sulfide control:*

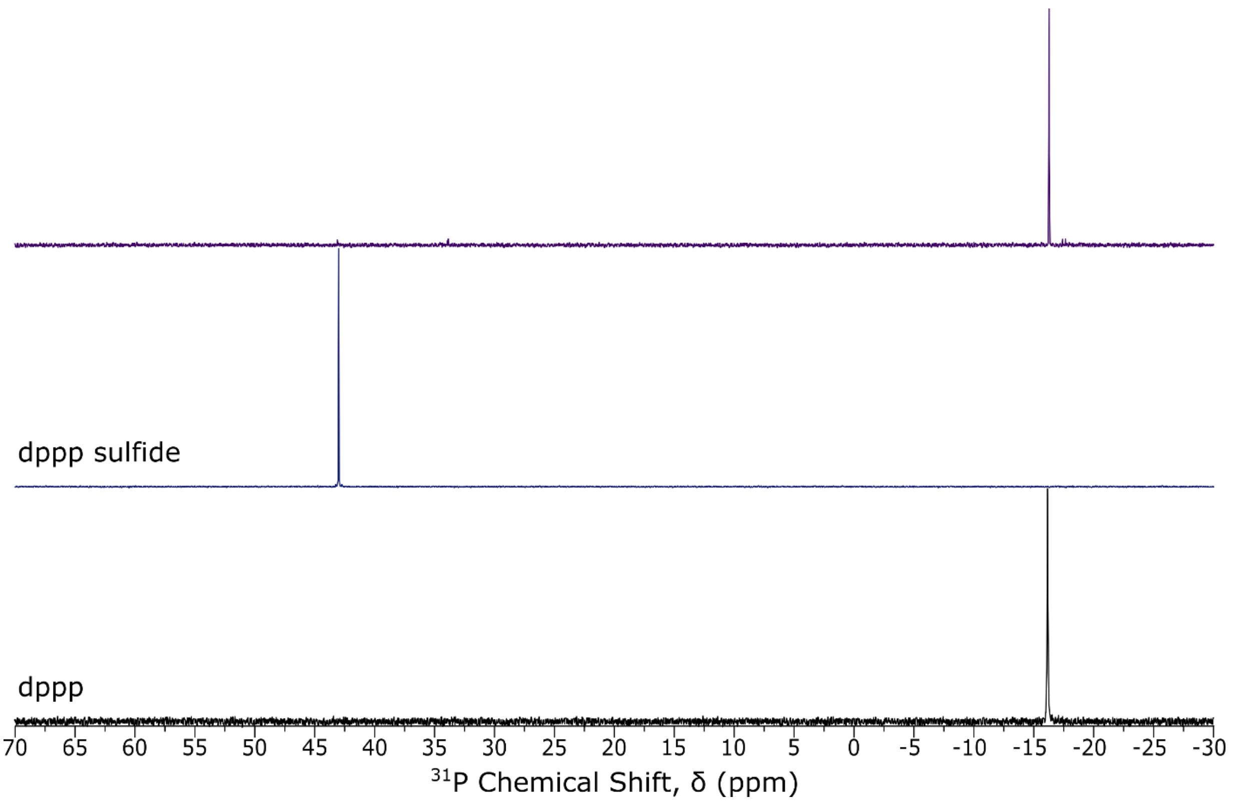


**Figure S3.** $^{31}P\{^{1}H\}$ NMR spectra of a control experiment of dppp and N,N-diethylthiourea, with references of dppp and dppp sulfide. The control experiment consisted of dppp and N,N-diethylthiourea in ODE in an NMR tube, heated at 195 °C for 1 hour. $^{31}P\{^{1}H\}$ δ (ppm) in chloroform-*d*: dppp -16.29; dppp sulfide 43.1; control experiment -16.29.

*Phosphine information:*

**Scheme S1.** Molecular structures of phosphines used in the study.

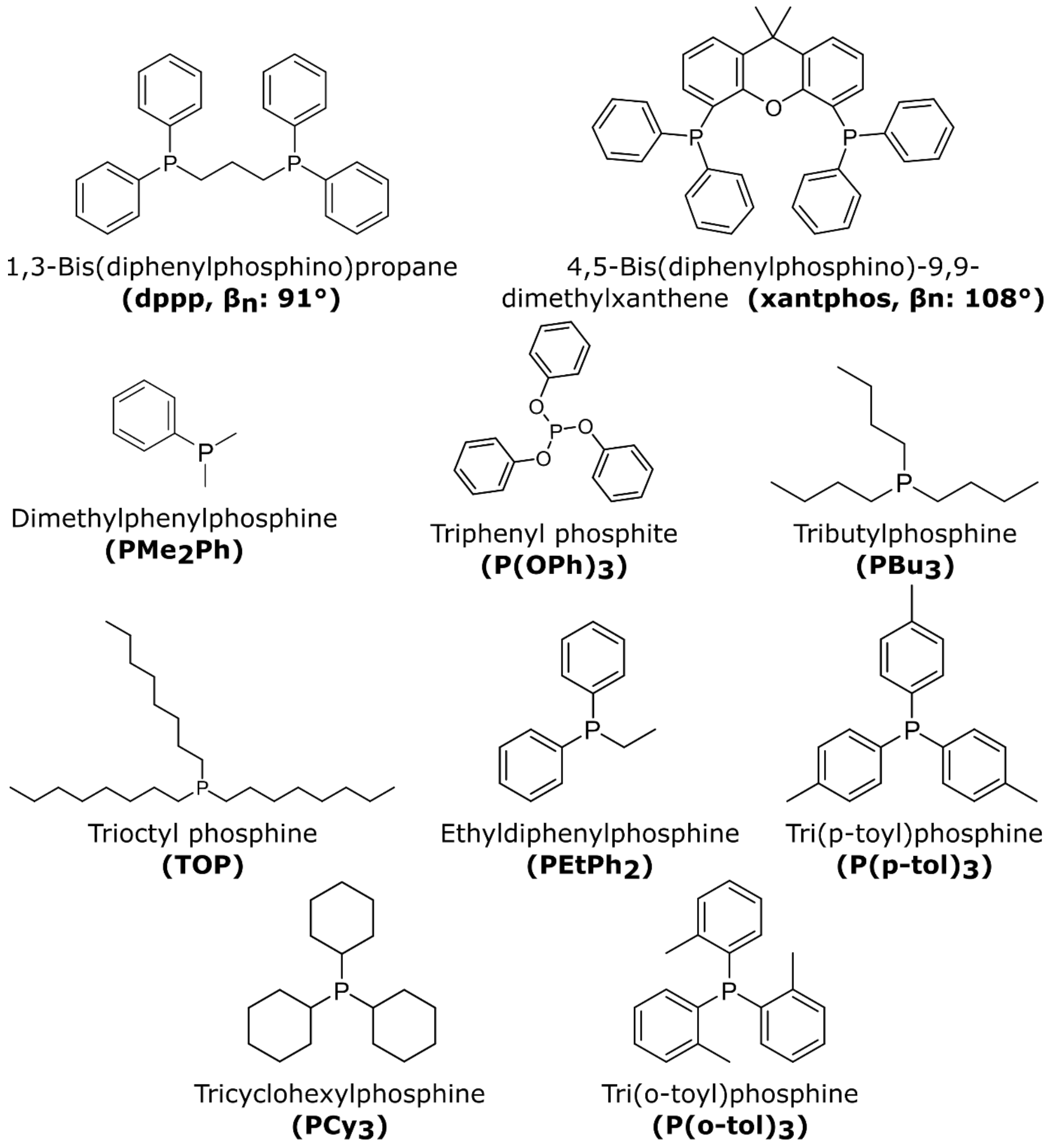


**Table S1.** Tolman electronic parameters (TEP) and cone angles of the monodentate phosphines.

| **Phosphine** | **TEP ($\nu_{CO}$ cm$^{-1}$)** | **Cone Angle (°)** |
|---|---|---|
| Dimethylphenylphosphine ($PMe_2Ph$)[a] | 2065 | 122 |
| Triphenylphosphite ($P(OPh)_3$)[a] | 2085 | 128 |
| Tributylphosphine ($PBu_3$)[a] | 2060 | 132 |
| Trioctylphosphine (TOP)[b] | 2061 | ~132 |
| Ethyldiphenylphosphine ($PEtPh_2$)[a] | 2066 | 140 |
| Tri-p-tolylphosphine ($P(p\text{-}tol)_3$)[a] | 2066 | 145 |
| Tricyclohexylphosphine ($PCy_3$)[a] | 2056 | 170 |
| Tri-o-tolylphosphine ($P(o\text{-}tol)_3$)[a] | 2084 | 194 |

[a]Tolman, C. A. *Chem Rev.* 1977.[1], [b]Sharp, C. G., *et al. Nano Lett.* 2020.[2]

*1 equivalent monodentate addition*

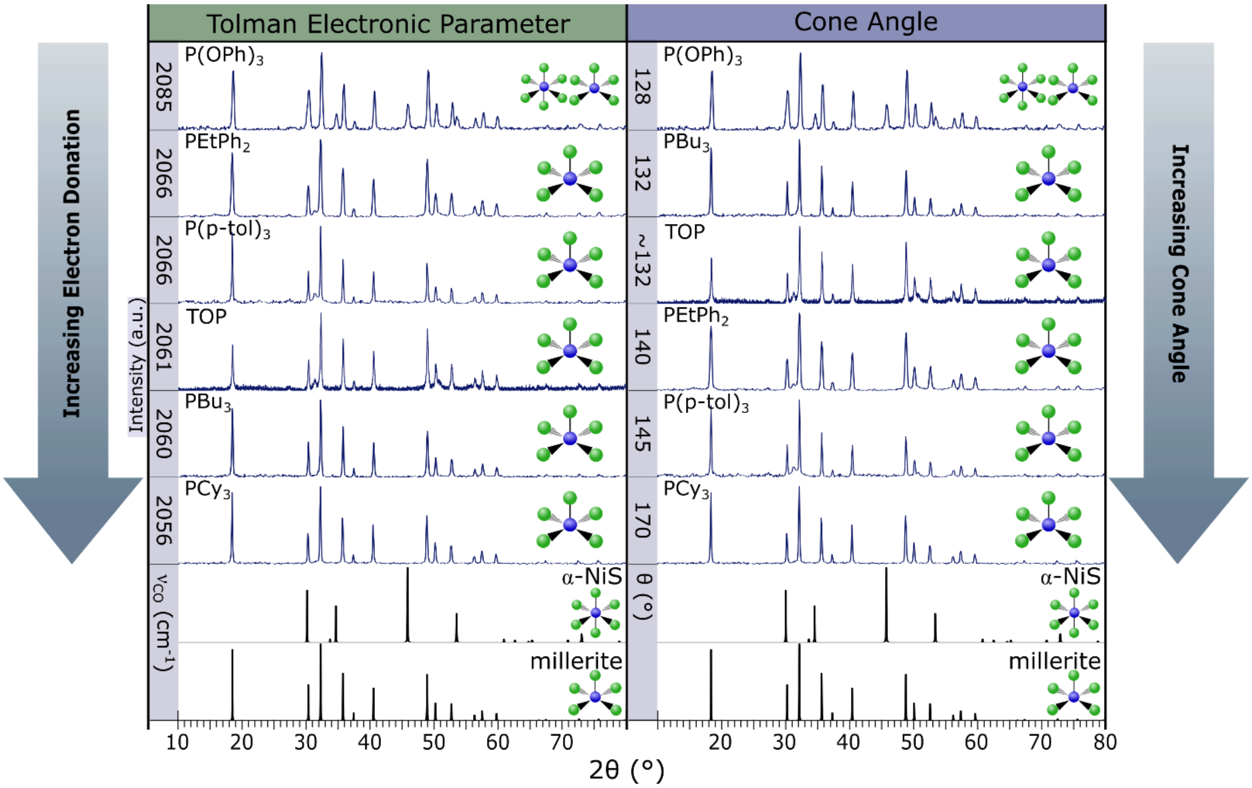


**Figure S4.** pXRD patterns for the products of the reaction of nickel(II) stearate and N,N-diethylthiourea at 195 °C in the presence of varying monodentate phosphines: $P(OPh)_3$, $PBu_3$, TOP, $PEtPh_2$, P(p-tol)$_3$, and $PCy_3$. When ordered via the phosphine electron donation ability (left) no trend is seen, but when ordered via cone angle (right) a trend can be seen, increasing the cone angle decreases the coordination number of the nickel sulfide produced. (α-NiS: 1010435 COD; millerite: 9004078 COD)

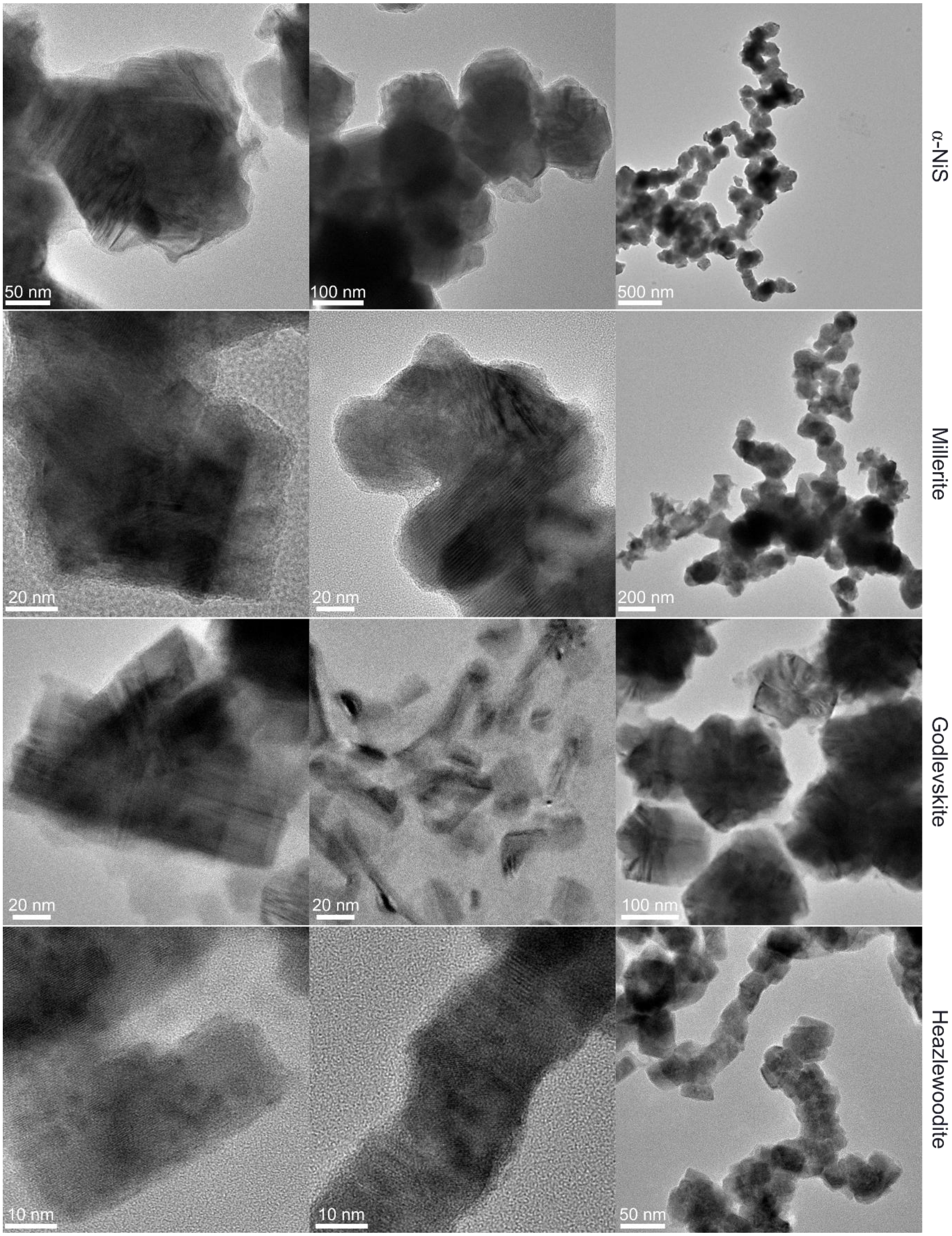


**Figure S5.** TEM images of phase pure samples. α-NiS was synthesized without phosphine. The imaged millerite was synthesized with two molar equivalents of $PBu_3$, godlevskite was synthesized with two molar equivalents of $PCy_3$, and heazlewoodite synthesized with four molar equivalents of $PEtPh_2$.

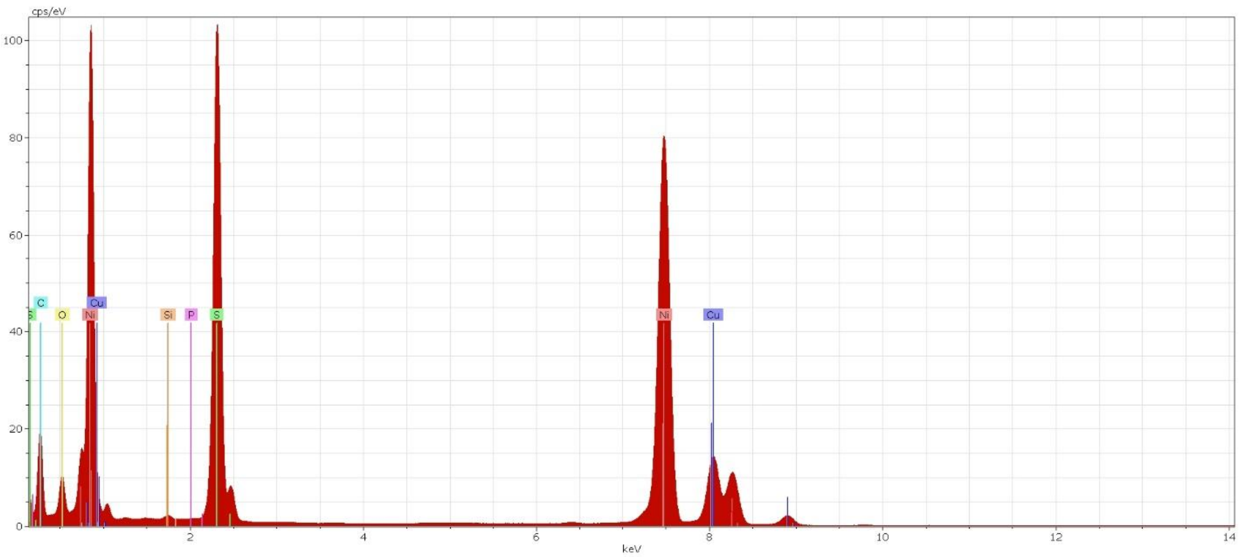


**Figure S6.** EDS spectrum of α-NiS was synthesized without phosphine **(TEM in Figure S5)**.

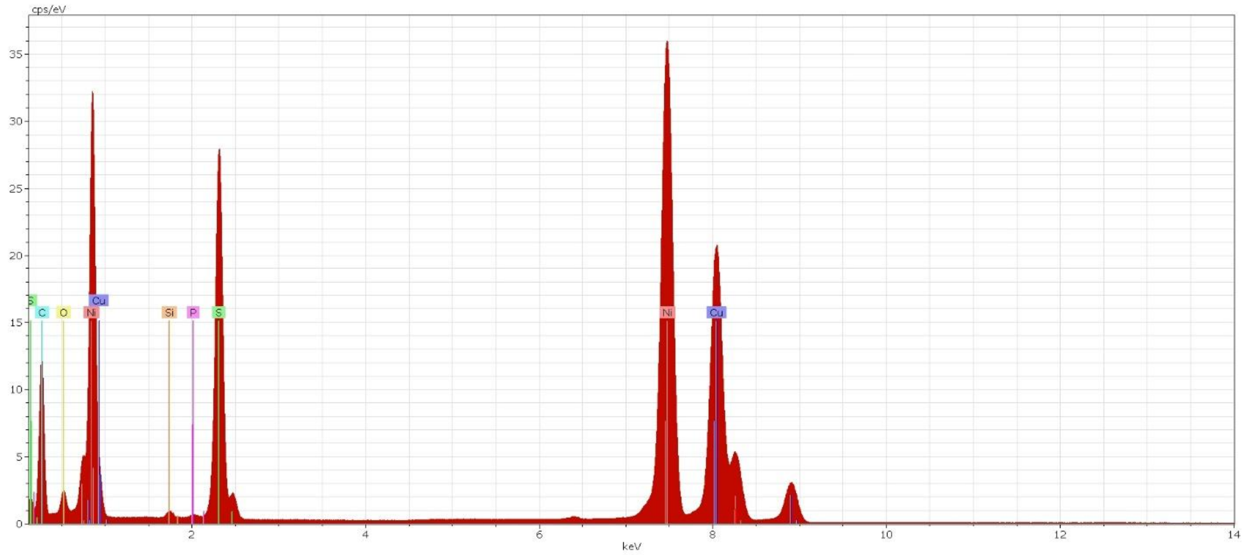


**Figure S7.** EDS spectrum of millerite, synthesized with two equivalents of $PBu_3$ **(TEM in Figure S5)**.

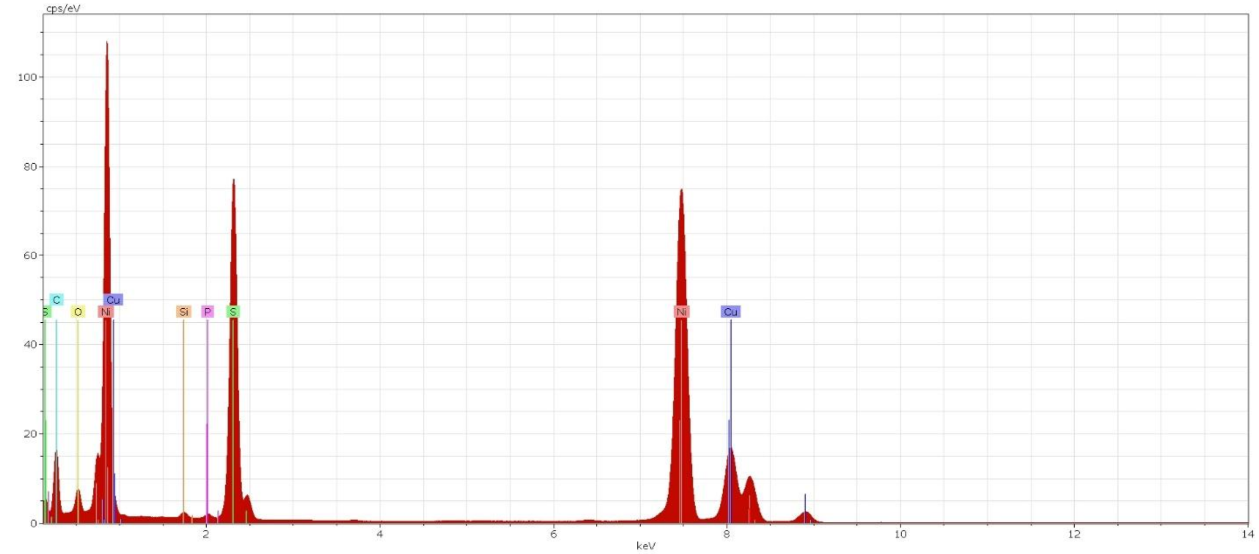


**Figure S8.** EDS spectrum of godlevskite, synthesized with two equivalents of $PCy_3$ **(TEM in Figure S5)**.

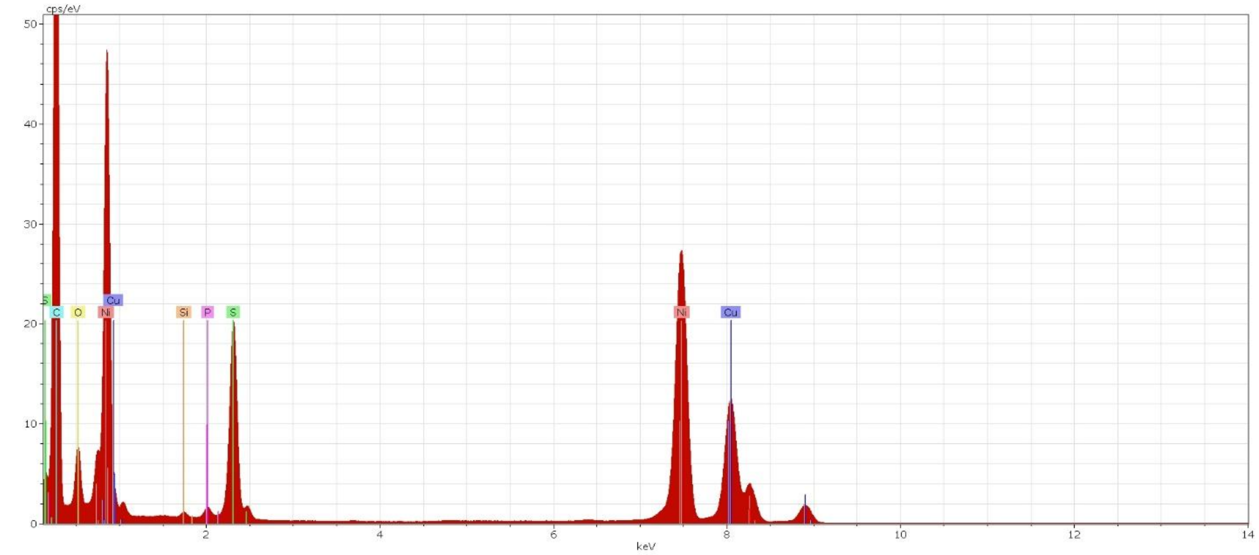


**Figure S9.** EDS spectrum of heazlewoodite, synthesized with four equivalents of $PEtPh_2$ **(TEM in Figure S5)**.

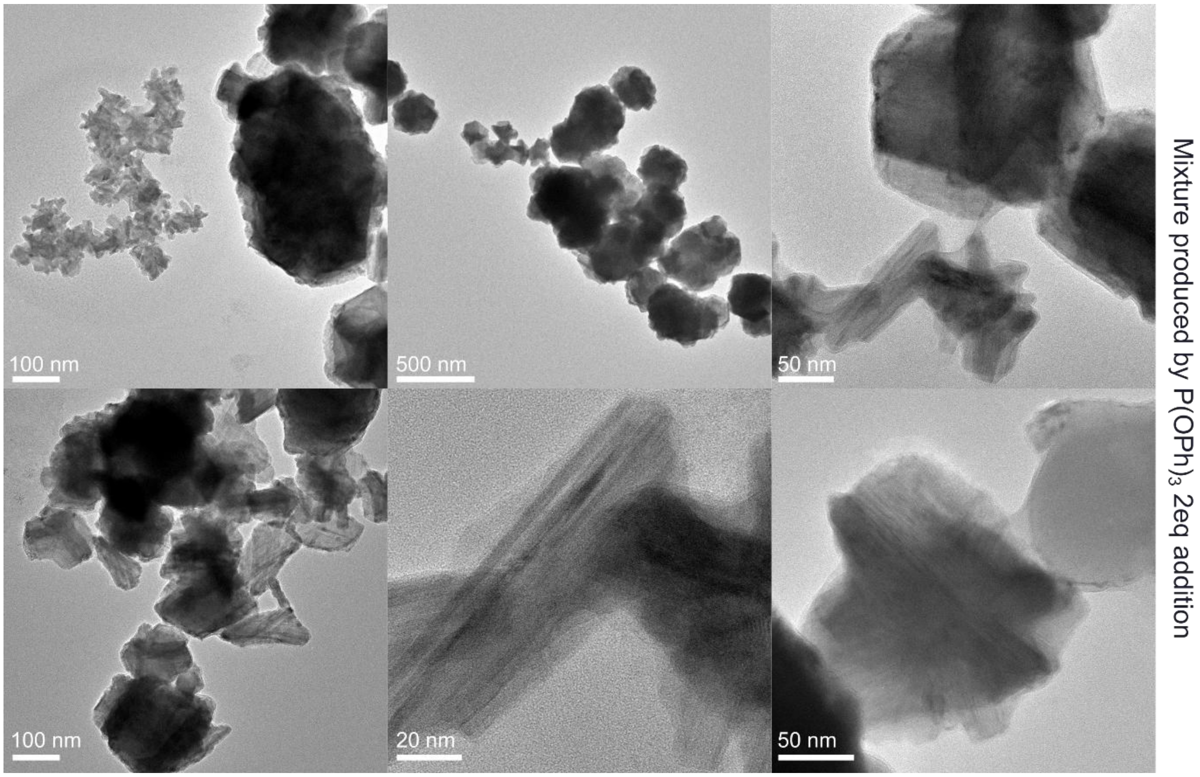


**Figure S10.** TEM images of mixed phase- α-NiS (CN6) and millerite (CN5)- products from synthesis with the addition of two molar equivalents of $P(OPh)_3$.

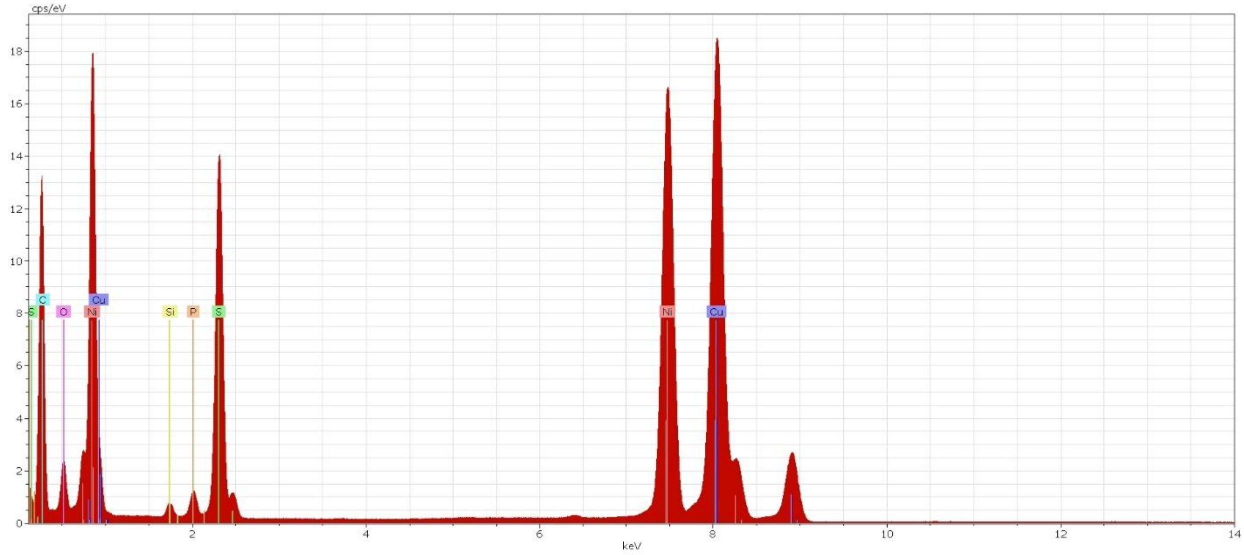


**Figure S11.** EDS spectrum of the mixed phase- α-NiS (CN6) and millerite (CN5)- product from the addition of two molar equivalents of $P(OPh)_3$ **(TEM in Figure S10)**.

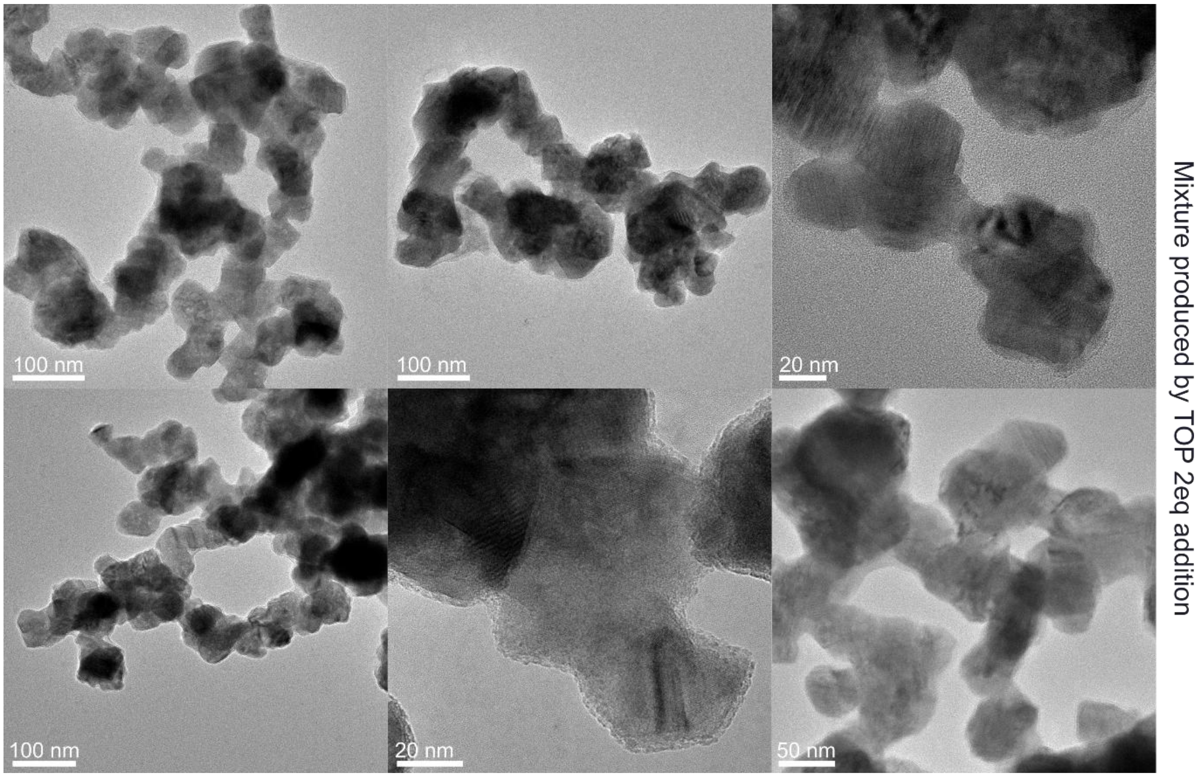


**Figure S12.** TEM images of mixed phase- millerite (CN5) and godlevskite (CN5/4)- products from synthesis with the addition of two molar equivalents of TOP.

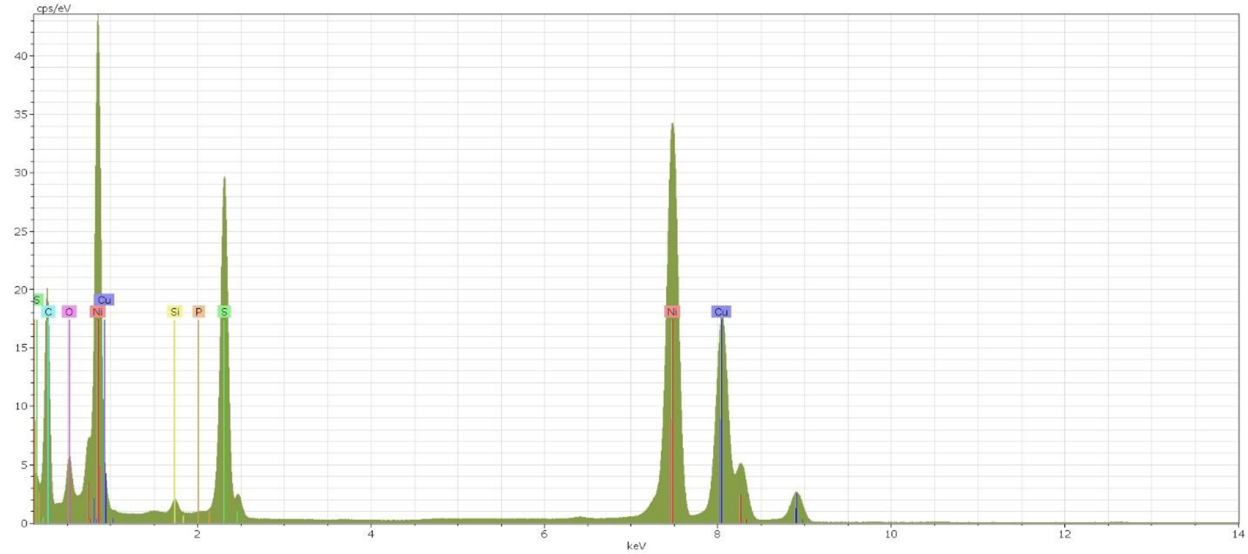


**Figure S13.** EDS spectrum of the mixed phase- millerite (CN5) and godlevskite (CN5/4)- product from the addition of two molar equivalents of TOP **(TEM in Figure S12)**.

All synthesized phases of nickel sulfide exhibit non-discrete shapes and have a tendency to stack as seen in **Figure S5, S10, and S12.** The mixed phase samples have no obvious differences in morphology **(Figure S10 and S12)**, but variation in size can be seen in the mixed phase sample from the reaction with the addition of $P(OPh)_3$ **(Figure S10)**. Because of stacking in all the samples, phase characterization from TEM is convoluted, so only crystalline size can be analyzed.

*2 equivalent monodentate addition*

**Table S2.** Rietveld refinements of products produced with varying cone angles (**Figure 4**)

| Phosphine | $PMe_2Ph$ | $P(OPh)_3$ | $PBu_3$ | TOP | $PEtPh_2$ | $P(p\text{-}tol)_3$ | $PCy_3$ |
|---|---|---|---|---|---|---|---|
| Phase | α-NiS* | α-NiS | | | | | |
| % | 63 ± 1 | 15 ± 0.5 | | | | | |
| Size (nm) | 46 ± 2 | 52 ± 11 | | | | | |
| Phase | Millerite | Millerite | Millerite | Millerite | | | |
| % | 37 ± 1 | 85 ± 1 | 100 ± 1 | 56 ± 2 | | | |
| Size (nm) | 38 ± 2 | 56 ± 1 | 46 ± 0.5 | 28 ± 0.5 | | | |
| Phase | | | | Godlevskite | Godlevskite | Godlevskite | Godlevskite |
| % | | | | 44 ± 3 | 100 ± 7 | 100 ± 4 | 100 ± 4 |
| Size (nm) | | | | 20 ± 2 | 24 ± 2 | 20 ± 2 | 34 ± 8 |
| Rwp % | 1.09 | 1.27 | 1.25 | 1.57 | 4.29 | 2.18 | 2.58 |
| $\chi^2$ | | | | | | | |

All refinements were performed with Rietveld methods, *except for the α-NiS in the synthesis with $PMe_2Ph$, which was performed with the Decomposition method.

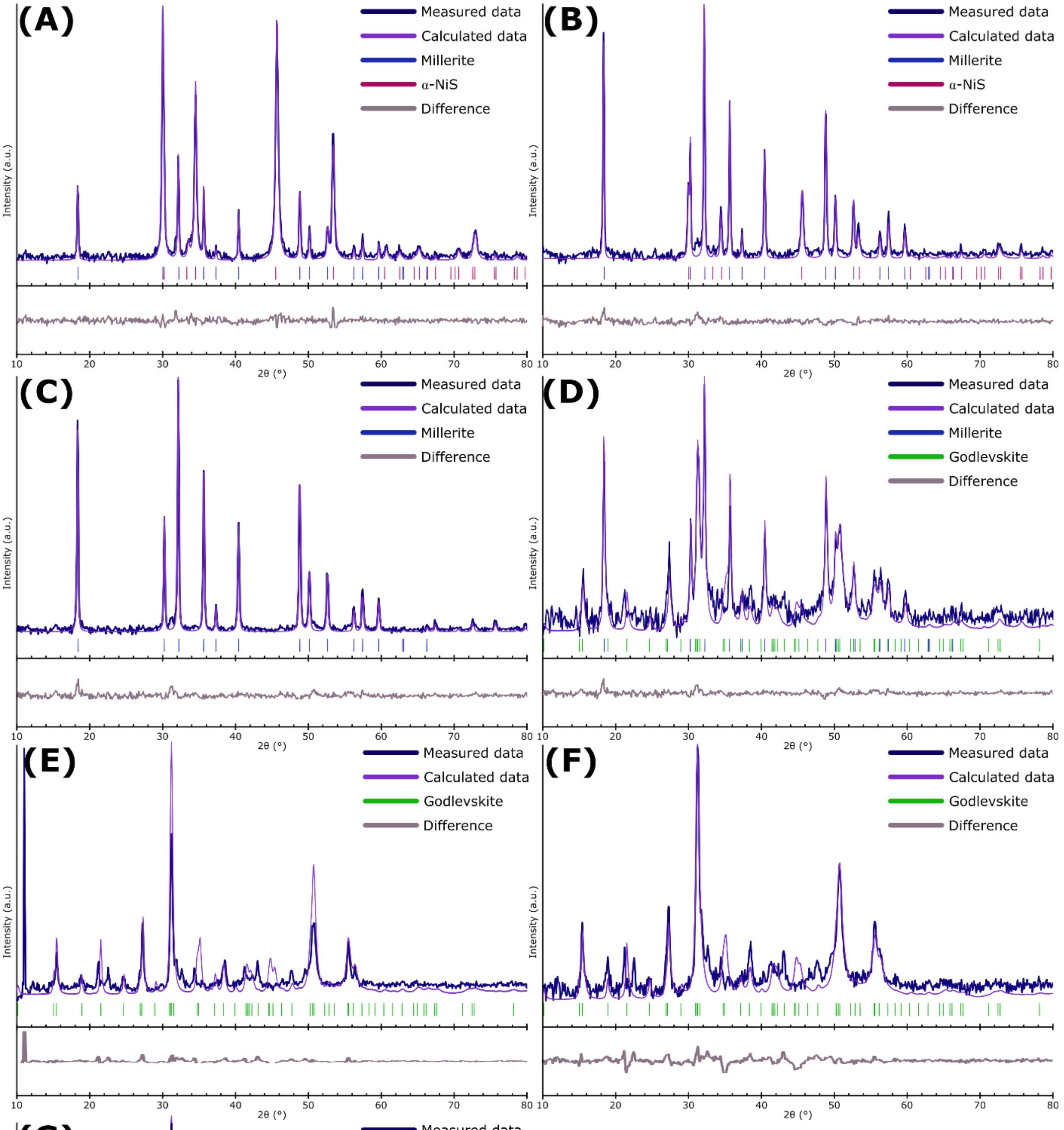


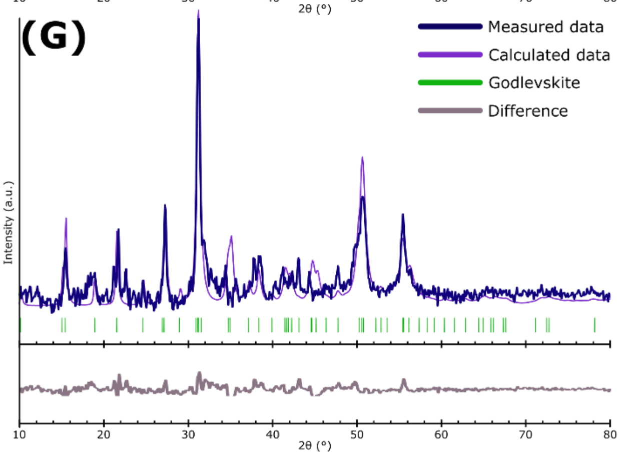


**Figure S14.** Phase refinement for reactions with 2 equivalents of (A) $PMe_2Ph$, (B) $P(OPh)_3$, (C) $PBu_3$, (D) TOP, (E) $PEtPh_2$, (F) $P(p\text{-tol})_3$, and (G) $PCy_3$. Refinements were performed using α-NiS (1010435 COD), millerite (9004078 COD), and godlevskite (9013880 COD).

From the Rietveld refinements performed on the cone angle study with two equivalent phosphine addition, a general trend in size can be seen **(Table S2)**. Syntheses with phosphines with smaller cone angles (122°-132°) produced nanoparticles that were ~40-50+ nm, while larger cone angles (132°-170°) produce smaller nanoparticles with sizes ~20-30 nm. For a single phase across multiple phosphines, no trend is observed in relation to phosphine cone angle and nanoparticle size.

*$P(o\text{-}tol)_3$ pXRD*

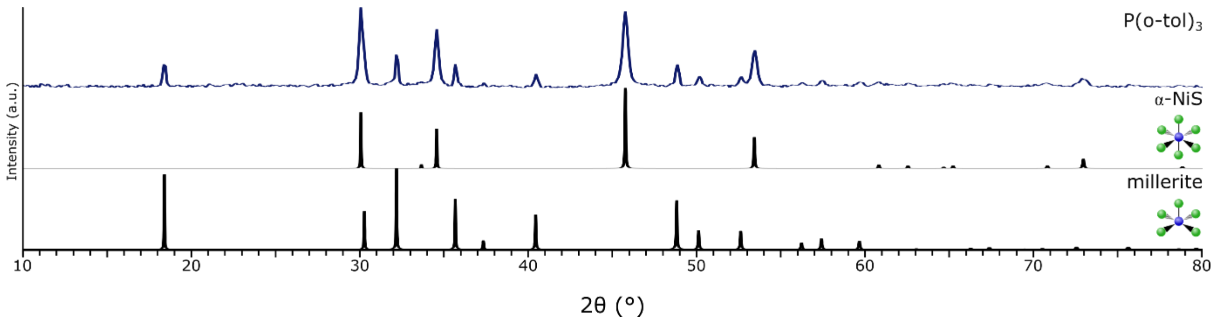


**Figure S15.** pXRD patterns for the product of the reaction of nickel(II) stearate with one equivalent of $P(o\text{-}tol)_3$. (α-NiS: 1010435 COD; millerite: 9004078 COD)

*UV-vis and extinction coefficient of $Ni(dppp)_2$*

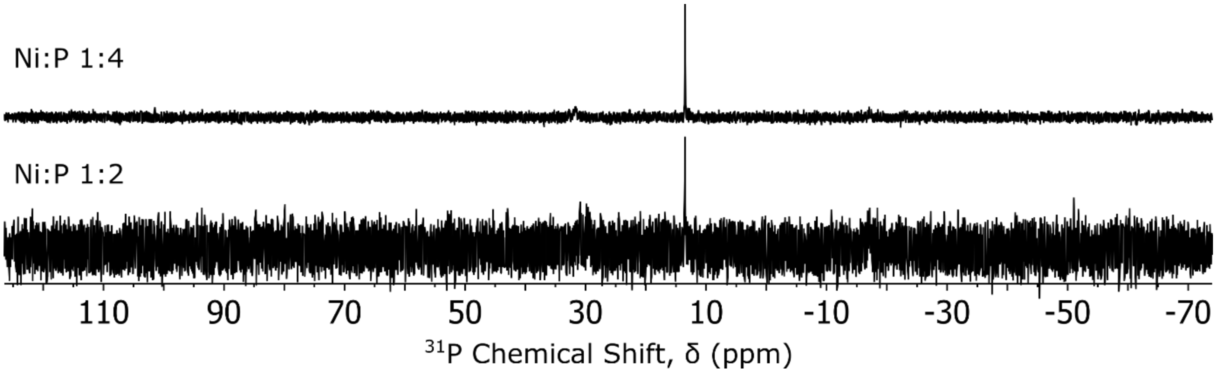


**Figure S16.** $^{31}P\{^1H\}$ spectra of reaction solutions of nickel(II) stearate and dppp in ODE heated to 170 °C with Ni:P ratios of 1:4 and 1:2. Chemical shift lines up with $Ni(dppp)_2$.[3,4] $^{31}P\{^1H\}$ δ (ppm) in benzene-*d6*: 13.49.

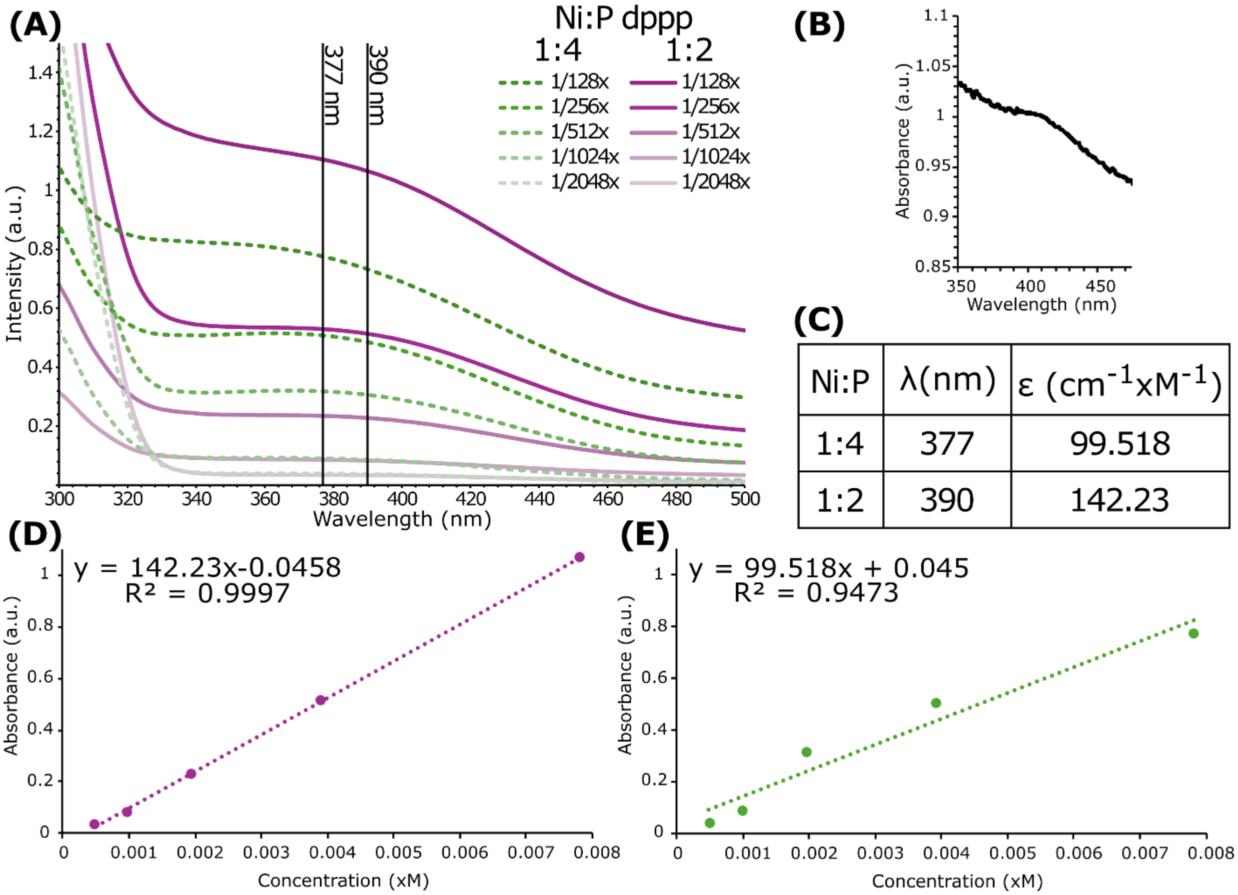


**Figure S17.** (A) UV-vis spectra for reaction solutions of nickel(II) stearate and dppp in ODE heated to 170 °C with Ni:P ratios of 1:4 (green, dotted line) and 1:2 (purple, solid line). ODE was used as the solvent. A "stock" solution of 2:1 reaction solution:ODE was made and labeled x, which was then serially diluted to collect the spectra shown above. (B) Normalized UV-vis of nickel(II) stearate in ODE. The solution was heated to dissolve the solid and the UV-vis taken at ~85 °C in a UV-vis with a heating stage. An absorbance peak was seen at 411 nm. (C) Absorbance peak wavelength and calculated extinction coefficients for both Ni:P solutions. (D&E) Absorbance vs. concentration for each Ni:P solution fitted with line equations for the determination of the extinction coefficients.

Note on calculated extinction coefficients:

$^{31}P$ NMR spectra of solutions of Ni:P 1:2 and 1:4 gave the same signal of a complex **(Figure S16)**, indicating the complex $Ni(dppp)_2$ is the only product. Therefore for the green solution of Ni:P 1:2 one expects ½ of the nickel to remain as nickel(II) stearate. The presence of and optical absorbtion of the nickel(II) stearate (B) convolutes the measurement of the extinciton coeffficient of $Ni(dppp)_2$; the apparent absorption maximum in UV-vis was at 390 nm with $\varepsilon = 142\ cm^{-1}M^{-1}$ **(Figure S17)**.

When nickel(II) stearate and dppp were combined in a Ni:P ratio of 1:4, the solution was orange-yellow with an absorption maximum of 477 nm. The measured extinction coefficient of $Ni(dppp)_2$ was $\varepsilon = 99\ cm^{-1}M^{-1}$. Because nickel(II) stearate was not present, this is the more accurate measurement of the extinction coefficient **(Figure S17)**.

Despite these differences, both Ni:P 1:4 and 1:2 solutions yield extinction coefficients suggesting a non-centrosymmetric d-d transition, consistent with a tetrahedral complex.

*$P(OPh)_3$ time study*

**Table S3.** Percent composition of millerite and α-NiS and refinement values for the pXRD patterns from products in **Figure 5**.

| Time | α-NiS (%) | Millerite (%) | Rwp (%) | $\chi^2$ |
|---|---|---|---|---|
| 1 min | 52 ± 3 | 48 ± 7 | 1.50% | 1.6247 |
| 5 min | 30.9* | 69 ± 5 | 1.46% | 1.4908 |
| 10 min | 22 ± 2 | 78 ± 3 | 1.37% | 1.3871 |
| 60 min | 4.6 ± 9 | 95 ± 15 | 1.16% | 1.5807 |

*RIR Method Quantitative analysis did not provide a significant standard deviation.

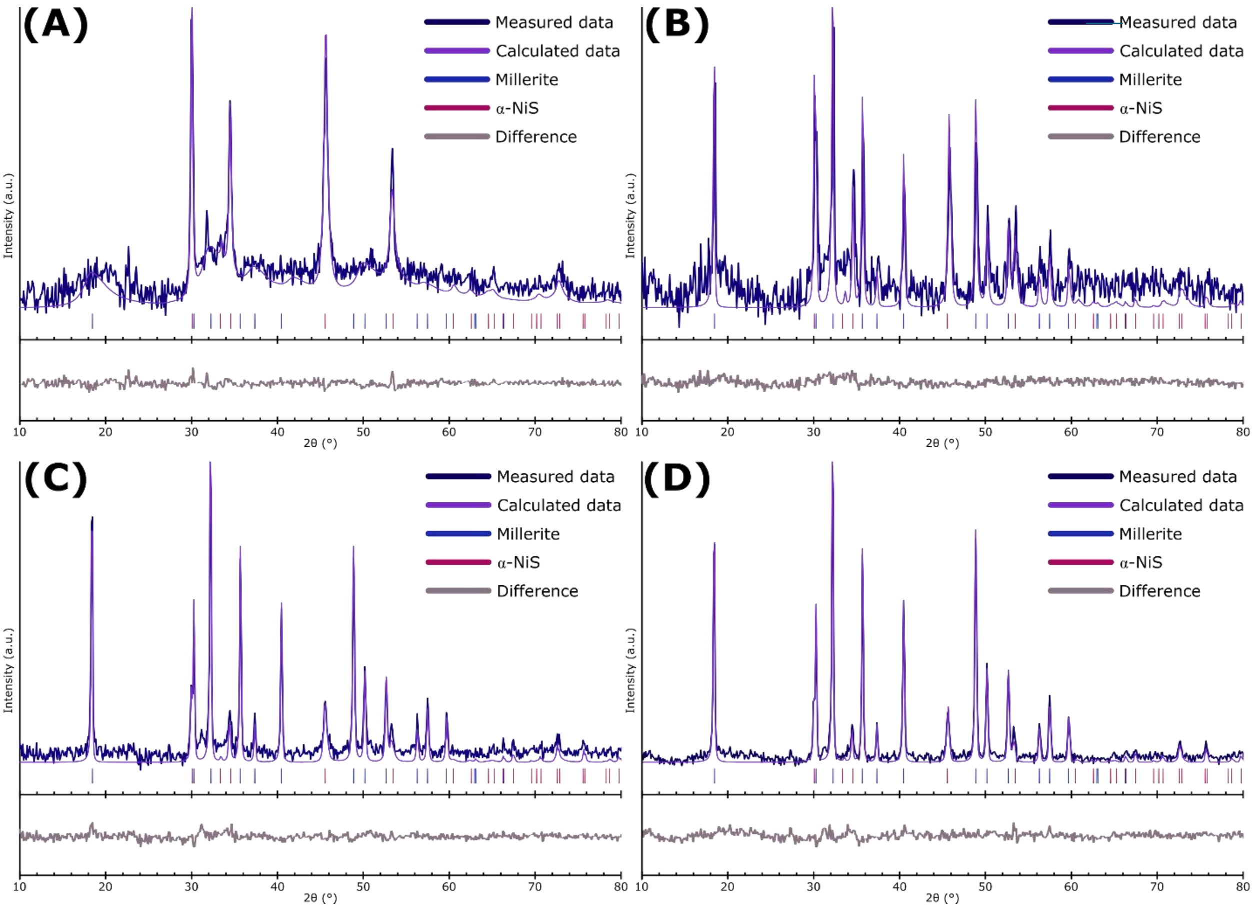


**Figure S18.** Phase refinement for (A) 1 min, (B) 5 min, (C) 10 min, and (D) 60 min reactions in $P(OPh)_3$ time study. Refinements were performed using millerite (9004078 COD) and α-NiS (1010435 COD).

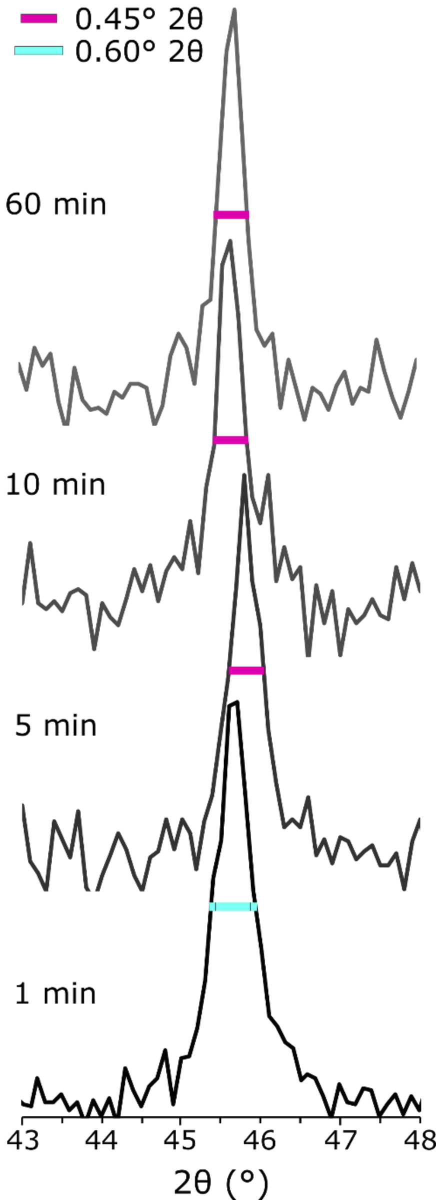


**Figure S19.** pXRD patterns of products from 1, 5, 10, and 60 minute reactions from **Figure 5** zoomed in on the α-NiS peak at 45.6-45.8° 2θ (patterns normalized to this peak). At 1 minute the full width half max is 0.60°, denoted with a light blue bar. At 5, 10, and 60 minutes the line width is 0.45°, denoted with a pink bar. Via the Scherrer equation, particles from the 1 minute were sized to 14.36 nm and 5, 10, and 60 minutes were sized to 19.15 nm. The length of the pink bar is marked with black lines within the blue bar in the 1 minute peak to better show the difference between the two.

*Density Functional Theory models*

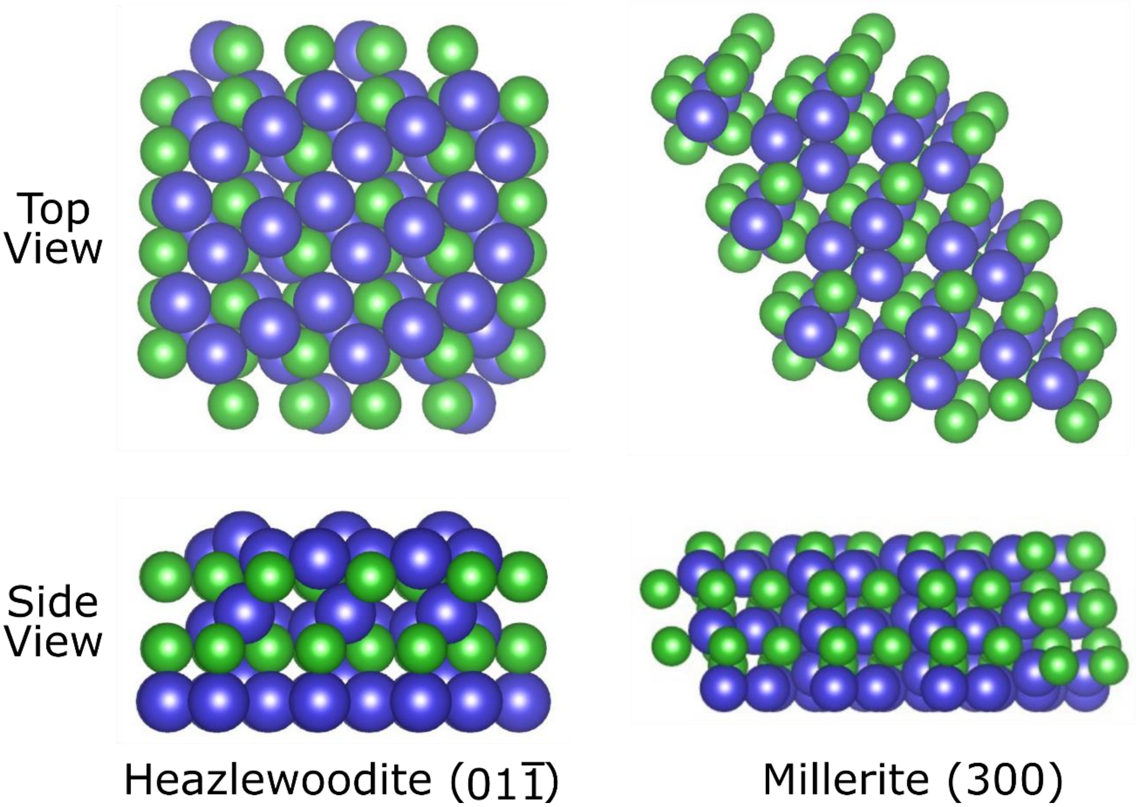


**Figure S20.** Top and side views of the clean surfaces of heazlewoodite (01$\bar{1}$) and millerite (003). Ni, blue; S, green.

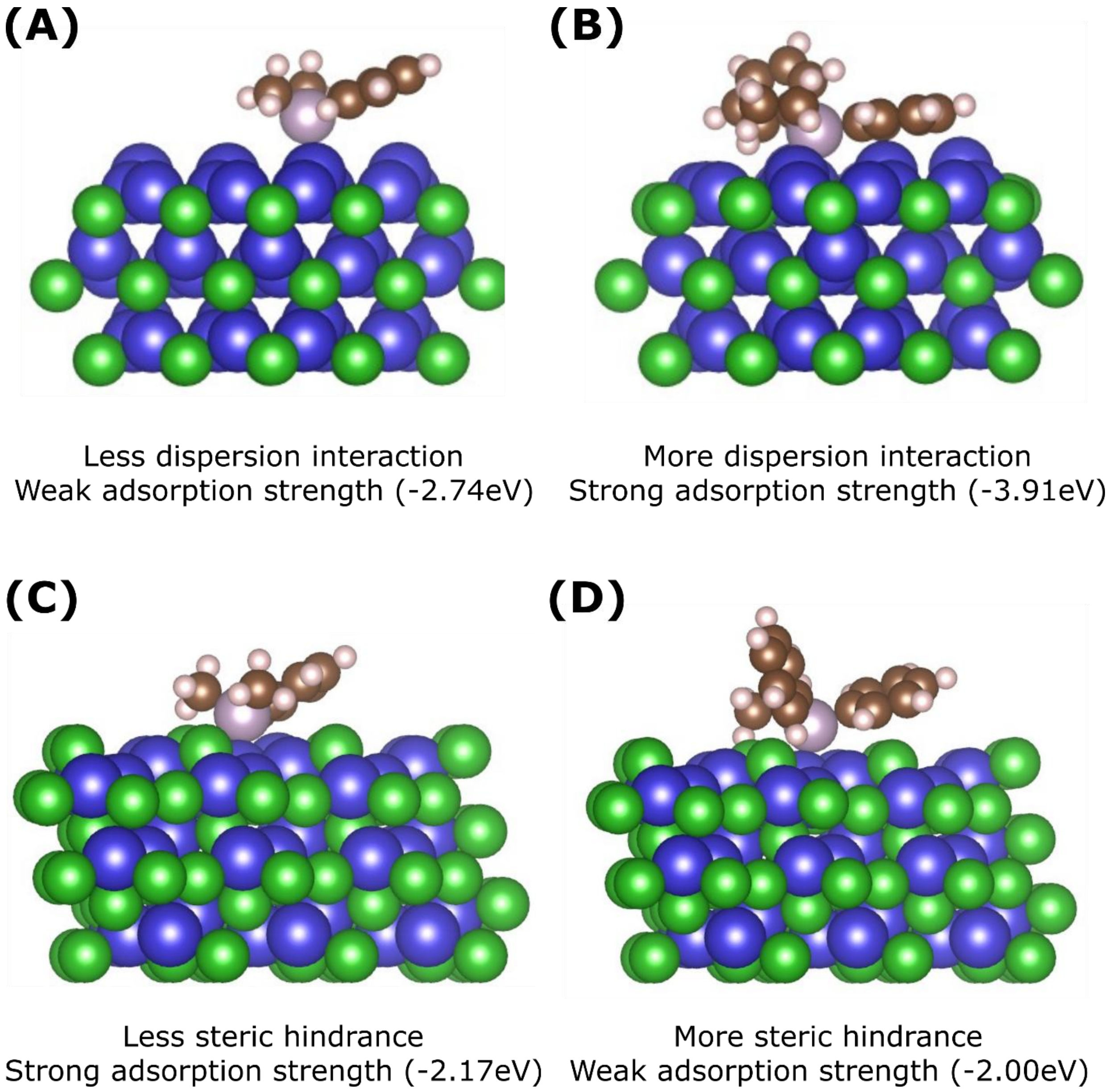


**Figure S21.** DFT-optimized adsorption structures and energies of phosphine ligands on heazlewoodite $(01\bar{1})$ and millerite (300) surfaces: (A) $PMe_2Ph$ on heazlewoodite; (B) $PEtPh_2$ on heazlewoodite; (C) $PMe_2Ph$ on millerite; (D) $PEtPh_2$ on millerite. Ni, blue; S, green; P, pale blue; C, brown; H, white.

**Table S4.** Summary table of syntheses that produce phase pure products. Throughout all syntheses nickel(II) stearate and N,N-diethylthiourea are used as the nickel and sulfur precursors, respectively.

| Phosphine | mmol of phosphine | mmol of nickel | mmol of sulfur | Phase synthesized | Cation CN |
|---|---|---|---|---|---|
| **-** | - | 1 | 6 | α-NiS | CN6 |
| **dppp** | 0.5 | 1 | 6 | Millerite | CN5 |
| | 1 | 1 | 6 | Millerite | CN5 |
| | 1 | 1 | 8 | Millerite | CN5 |
| | 1 | 1 | 16 | Millerite | CN5 |
| | 1 | 1 | 2 | Heazlewoodite | CN4 |
| | 2 | 1 | 6 | Heazlewoodite | CN4 |
| | 1 | 1 | 4 | Godlevskite | CN5/4 |
| **xantphos** | 0.5 | 1 | 6 | Millerite | CN5 |
| | 1 | 1 | 6 | Millerite | CN5 |
| | 2 | 1 | 6 | Millerite | CN5 |
| | 1 | 1 | 1 | Heazlewoodite | CN4 |
| | 1 | 1 | 2 | Godlevskite | CN5/4 |
| | 1 | 1 | 4 | Godlevskite | CN5/4 |
| **$PEtPh_2$** | 1 | 1 | 6 | Millerite | CN5 |
| | 1 | 1 | 1 | Heazlewoodite | CN4 |
| | 1 | 1 | 2 | Godlevskite | CN5/4 |
| | 2 | 1 | 6 | Godlevskite | CN5/4 |
| **$PBu_3$** | 1 | 1 | 6 | Millerite | CN5 |
| | 2 | 1 | 6 | Millerite | CN5 |
| **TOP** | 1 | 1 | 6 | Millerite | CN5 |
| **P(p-tol)$_3$** | 1 | 1 | 6 | Millerite | CN5 |
| | 2 | 1 | 6 | Godlevskite | CN5/4 |
| **$PCy_3$** | 1 | 1 | 6 | Millerite | CN5 |
| | 2 | 1 | 6 | Godlevskite | CN5/4 |
| **Two-step:** | | | | | |
| **$PEtPh_2$** | 1 | α-NiS or millerite nanoparticles | - | Heazlewoodite | CN4 |
| **$PBu_3$** | 1 | millerite | - | Heazlewoodite | CN4 |

**References**


(1) Tolman, C. A. Steric Effects of Phosphorus Ligands in Organometallic Chemistry and Homogeneous Catalysis. *Chem Rev* 1977, *77* (3), 313–348. https://doi.org/10.1021/cr60307a002.

(2) Sharp, C. G.; Leach, A. D. P.; Macdonald, J. E. Tolman's Electronic Parameter of the Ligand Predicts Phase in the Cation Exchange to CuFeS2 Nanoparticles. *Nano Lett* 2020, *20* (12), 8556–8562. https://doi.org/10.1021/acs.nanolett.0c03122.

(3) Fisher, K. J.; Alyea, E. C. Metal Vapour Synthesis of Zero-Valent Nickel Phosphine Complexes and Their Characterization by 31P NMR Spectroscopy. *Polyhedron* 1989, *8* (1), 13–15. https://doi.org/10.1016/S0277-5387(00)86371-8.

(4) Uvarov, V.; Popov, I. Metrological Characterization of X-Ray Diffraction Methods for Determination of Crystallite Size in Nano-Scale Materials. *Mater Charact* 2007, *58* (10), 883–891. https://doi.org/10.1016/j.matchar.2006.09.002.
